# Identification-Based Comparative Proteomic and Peptidomic Profiling of *Vespa mandarinia* and *Apis mellifera* Venoms Supported by De Novo Transcriptomic Annotation

**DOI:** 10.3390/toxins18080351

**Published:** 2026-08-17

**Authors:** Lanfen Yang, Li Li, Jinwei Dao, Li Yang, Qi Yang

**Affiliations:** 1 Dehong Biomedical Engineering Research Center, Dehong Normal College, Dehong 678400, China; lanfenyang2011@163.com (L.Y.);; 2 Institute of Medical Technology, Dehong Vocational College, Dehong 678400, China

**Keywords:** *Vespa mandarinia*, *Apis mellifera*, venom, proteomics, peptidomics, transcriptome, LC-MS/MS, venomomics

## Abstract

Hymenopteran venoms contain diverse proteins and peptides that shape envenomation, defense, predation, and allergic responses. Honeybee venom from *Apis mellifera* is well characterized, while molecular resources for the Asian giant hornet *Vespa mandarinia* remain less curated. We compared protein- and peptide-fraction LC-MS/MS identification datasets from *V. mandarinia* venom (VM-V) and *A. mellifera* venom (AM-V), supported by a de novo *V. mandarinia* transcriptome-derived database. Protein-level identification yielded 197 protein groups in VM-V PRO and 164 protein groups in AM-V PRO. AM-V contained well-recognized honeybee venom components, including phospholipase A2, hyaluronidase, venom acid phosphatase, venom dipeptidyl peptidase 4, melittin precursor, mast cell degranulating peptide precursor, secapin, and allergen Api m 6. VM-V PRO contained transcriptome-supported candidate venom-associated proteins, including venom dipeptidyl peptidase 4-like, hyaluronidase-like, venom allergen 5-like, serine protease-like, apolipophorin-like, and hexamerin-like entries. Peptide-fraction annotation was strongest in AM-V PEP, led by melittin precursor, whereas VM-V PEP remained largely unannotated. The study focuses on identification, annotation, and hypothesis-generating functional summaries, not replicate-level differential abundance. GO, KEGG, and STRING analyses organized functional annotation patterns and prioritized candidate protein groups. These data provide an identification-based comparative venomomics resource for VM-V and AM-V and a foundation for targeted validation of candidate *V. mandarinia* venom-associated components.

## 1. Introduction

Hymenopteran venoms are complex secretions composed of enzymes, peptides, allergens, and other bioactive molecules [1]. These venoms mediate defense and predation, and sting exposure can cause local pain, systemic toxic effects, and allergic reactions [2]. Molecular characterization of venom proteins and peptides has therefore become central to toxinology, allergy research, and venom evolution studies [1].

Honeybee venom from *Apis mellifera* is among the most extensively studied Hymenoptera venoms. Its mature molecular repertoire includes enzymatic proteins, allergenic proteins, and short bioactive peptides, with melittin representing a dominant peptide component [3]. This well-curated venom system provides a useful comparator for evaluating LC-MS/MS venom fractionation and annotation because many honeybee venom proteins and peptides are represented in public databases and prior proteomic studies.

Hornet and wasp venoms are also rich sources of enzymes, allergens, and bioactive peptides. Reviews of wasp venom composition describe phospholipases, hyaluronidases, antigen 5-like proteins, proteases, and low-molecular-weight peptides as recurring functional classes [4]. These molecular features make *Vespa* venoms suitable for comparative venomomics, especially when protein-level and peptide-level data can be interpreted in parallel.

*Vespa mandarinia*, the Asian giant hornet, is of particular biological and medical interest because of its large body size, potent sting, ecological role, and expanding venom-relevant molecular resources. A de novo *V. mandarinia* transcriptome provided early gene-level annotation and SSR resources for this species [5], and a chromosome-level genome has recently expanded the reference framework for genome-informed analysis [6].

These advances create a clear context for comparative datasets that place *V. mandarinia* venom alongside a well-characterized honeybee venom comparator. Such comparisons help define annotation strengths, candidate protein families, and database-dependent interpretation. This is especially relevant for non-model venomous insects, where transcriptome-derived or genome-informed databases can expand the searchable protein space and reduce annotation loss [7].

**Figure 1:**
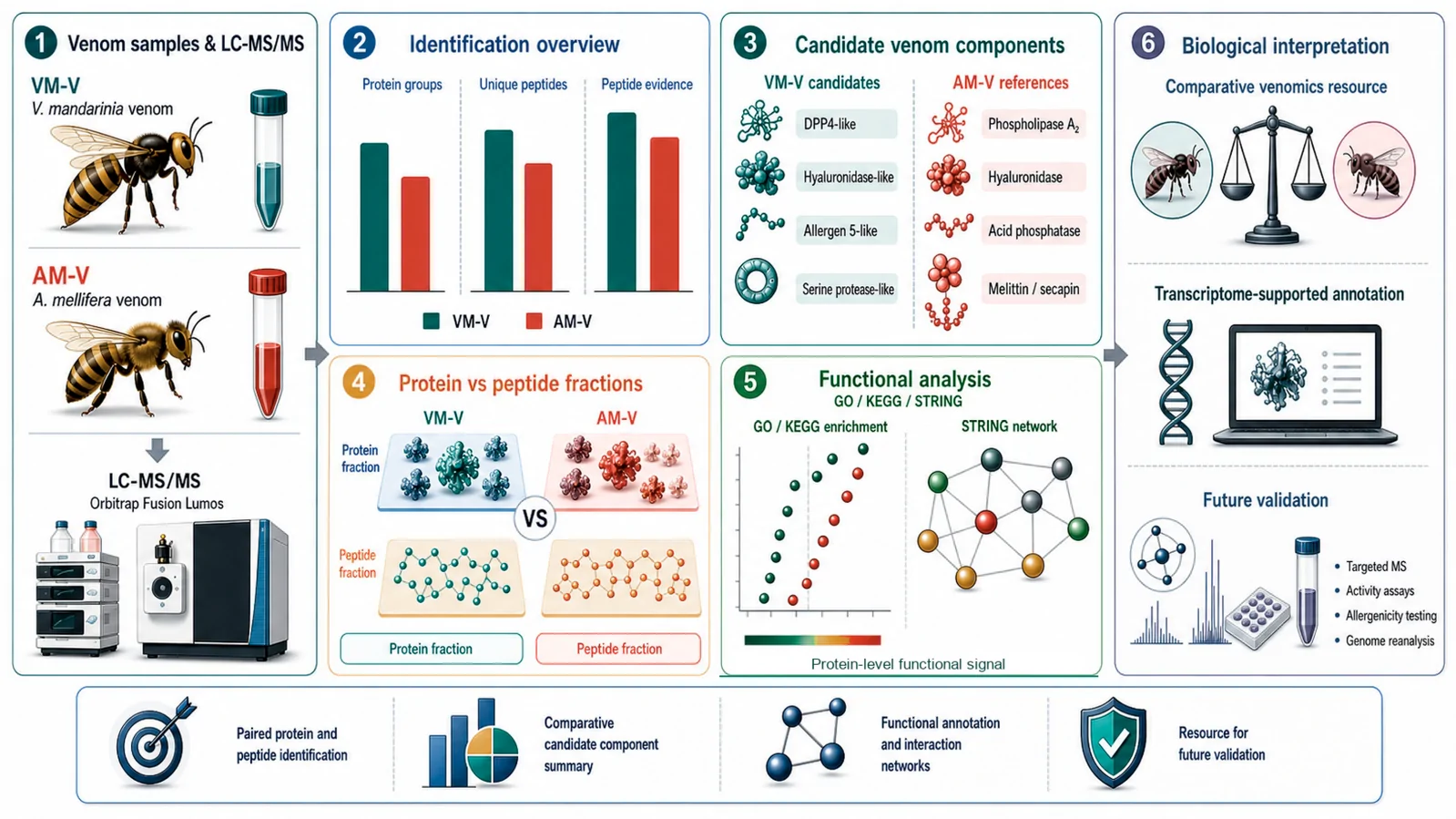
Study overview and analytical framework. Graphical overview of protein- and peptide-fraction LC-MS/MS identification, candidate venom-component screening, and GO, KEGG, and STRING annotation. The numbered panels indicate the workflow sequence; arrows indicate analytical flow; dashed separators distinguish subcomponents within a panel; and the color scheme distinguishes VM-V, AM-V, protein-fraction, peptide-fraction, and functional-analysis elements. VM-V protein identification used a de novo transcriptome-derived database, whereas AM-V used the *A. mellifera* reference protein database. VM-V, *Vespa mandarinia* venom; AM-V, *Apis mellifera* venom; LC-MS/MS, liquid chromatography-tandem mass spectrometry; GO, Gene Ontology; KEGG, Kyoto Encyclopedia of Genes and Genomes; STRING, Search Tool for the Retrieval of Interacting Genes/Proteins.

In this study, we analyzed existing protein- and peptide-fraction LC-MS/MS identification datasets for *V. mandarinia* venom (VM-V) and *A. mellifera* venom (AM-V). We used the accompanying *V. mandarinia* transcriptome resource to support VM-V protein identification and integrated protein identification, peptide evidence, GO/KEGG annotation summaries, and STRING network outputs. The study provides an identification-based comparative venomomics resource focused on candidate venom-associated proteins, peptide-fraction annotation, and transcriptome-supported interpretation. The overall study design and analytical framework are summarized in Figure 1.

## 2. Results

### 2.1. Overview of Venom Fractionation and LC-MS/MS Datasets

Four identification datasets were generated from the analyzed venom materials: VM-V PRO and AM-V PRO for the protein fractions, and VM-V PEP and AM-V PEP for the peptide fractions. Their fraction types and database-search configurations are summarized in Table 1.

### 2.2. Protein-Level Identification Profiles of VM-V and AM-V

**Figure 2:**
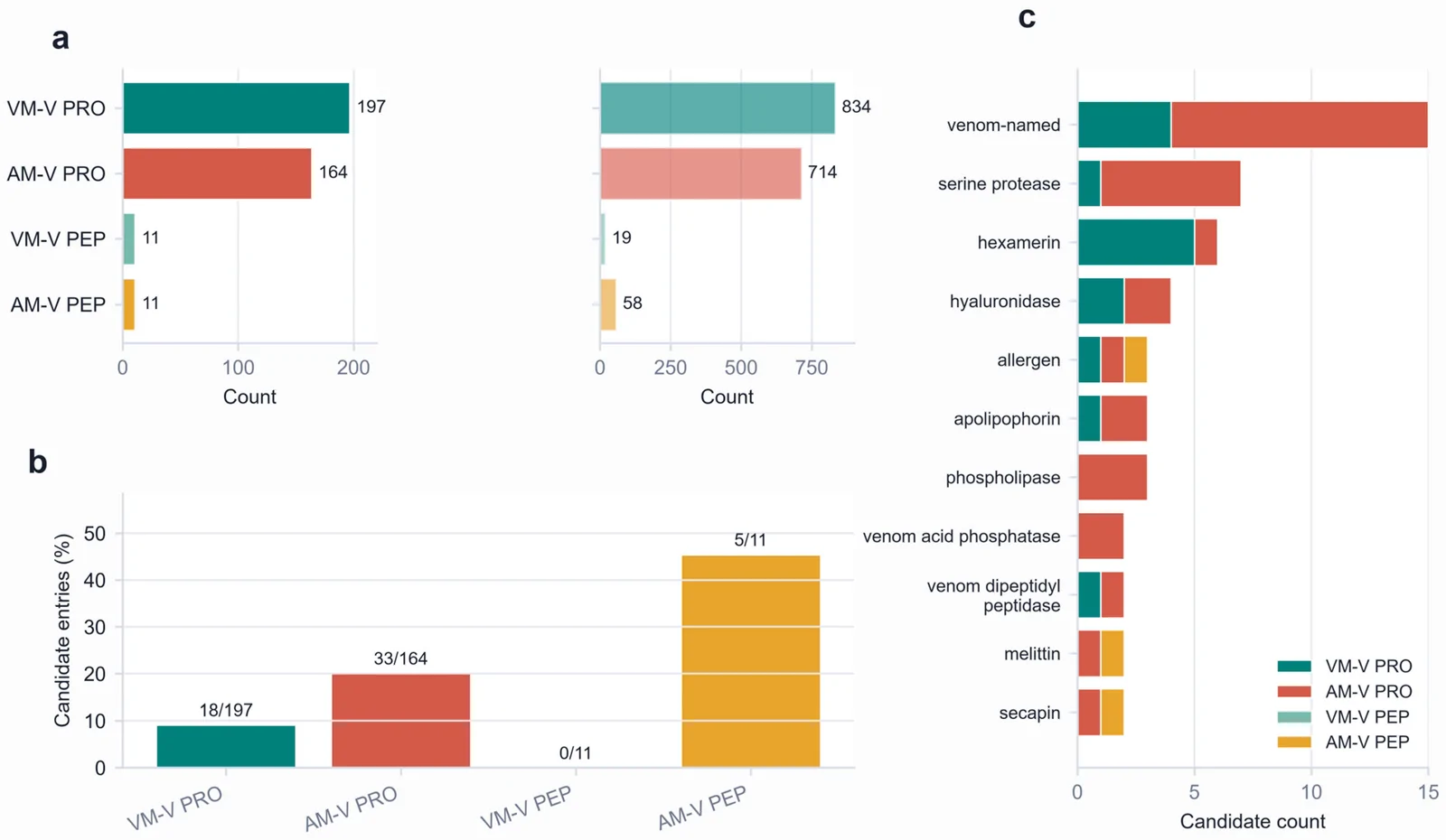
Identification scale and venom-associated candidate overview across VM-V and AM-V fractions. (**a**) Protein-group counts and unique peptide counts for VM-V PRO, AM-V PRO, VM-V PEP, and AM-V PEP. (**b**) Proportion of keyword-screened venom-associated candidate entries in each dataset; labels indicate candidate entries over total protein groups. (**c**) Distribution of major candidate categories detected by keyword screening across the four datasets. Candidate screening used venom-relevant protein and peptide terms. VM-V, *Vespa mandarinia* venom; AM-V, *Apis mellifera* venom; PRO, protein fraction; PEP, peptide fraction.

Protein-level analysis identified 197 protein groups in VM-V PRO and 164 in AM-V PRO (Figure 2a; Table 2). The corresponding peptide evidence and per-entry identification summaries are reported in Table 2. These metrics describe identification support and were not used as abundance measurements or differential statistics. Detailed protein-identification records are provided in Supplementary Table S1, and peptide-identification row counts are provided in Supplementary Table S2.

Transcriptome-supported annotation assigned protein names to 171 of 197 VM-V PRO entries. High-peptide-count VM-V PRO entries include apolipophorin-like protein (65 peptides), venom dipeptidyl peptidase 4-like (28 peptides), several hexamerin-like entries (27, 23, and 16 peptides), hyaluronidase-like entries (17 and 13 peptides), transferrin-like (14 peptides), venom allergen 5-like (eight peptides), and lysozyme-like proteins (Table 3). This protein set outlines a VM-V identification profile containing enzymes, allergen-like proteins, transport-related proteins, and other transcriptome-supported candidate venom-associated proteins. The complete keyword-screening results and dataset-level candidate summary are provided in Supplementary Tables S3 and S4.

**Figure 3:**
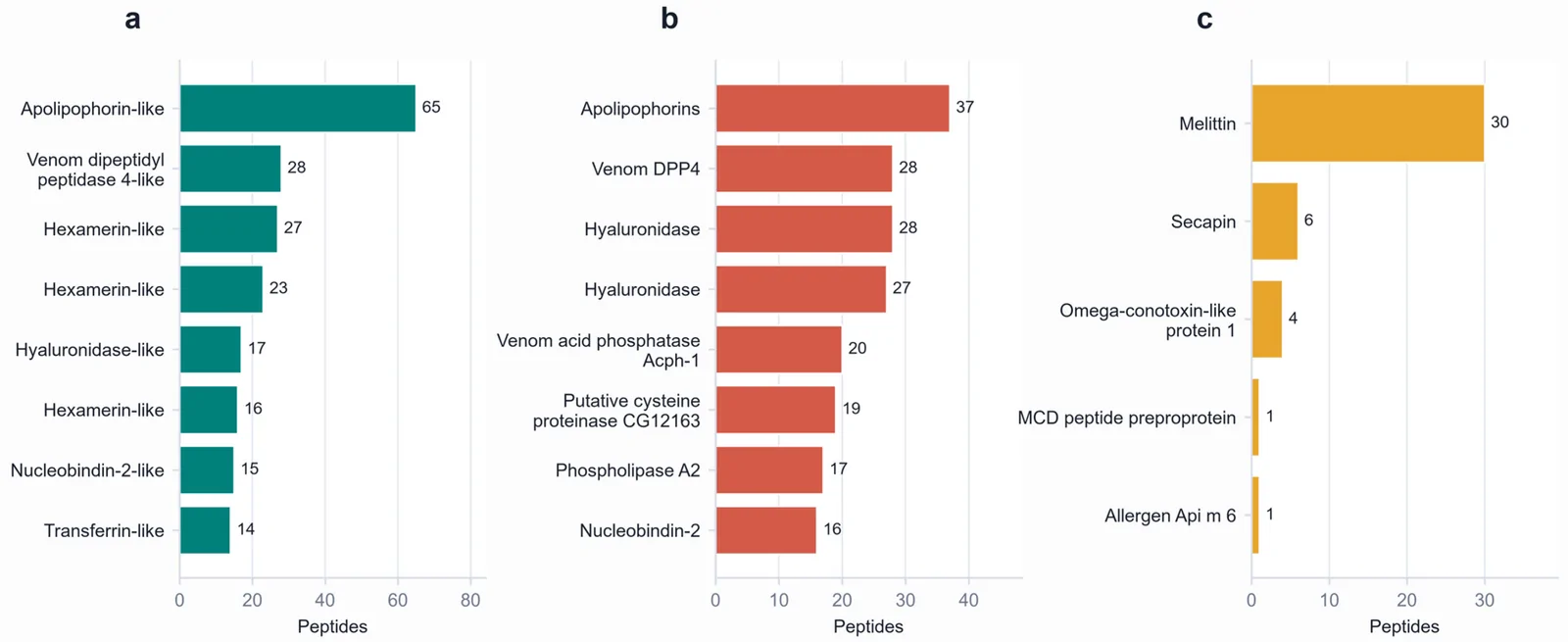
Representative candidate proteins and peptides detected in VM-V and AM-V. Candidate entries are ranked by peptide count. (**a**) Representative VM-V PRO candidates. (**b**) Representative AM-V PRO candidates. (**c**) AM-V PEP candidates; VM-V PEP did not yield named venom-peptide candidates under this annotation strategy. Colors indicate the dataset: teal, VM-V PRO; red, AM-V PRO; and gold, AM-V PEP. VM-V, *Vespa mandarinia* venom; AM-V, *Apis mellifera* venom; PRO, protein fraction; PEP, peptide fraction.

AM-V PRO shows the expected signature of honeybee venom protein identification. Detected entries include phospholipase A2 precursor (17 peptides), venom acid phosphatase Acph-1 isoforms (20 and seven peptides), hyaluronidase isoform X1 and hyaluronidase precursor entries (28 and 27 peptides), venom dipeptidyl peptidase 4 (28 peptides), venom carboxylesterase-6, venom serine proteases, allergen Api m 6, melittin precursor, mast cell degranulating peptide preproprotein, and secapin. Identification of these established honeybee venom proteins supports AM-V as a curated comparator for VM-V interpretation. Keyword screening identifies 33 venom-related candidate entries in AM-V PRO and 18 in VM-V PRO, reflecting the richer curated annotation available for honeybee venom and the value of transcriptome-supported annotation for VM-V (Figure 2b,c and Figure 3a,b).

### 2.3. Peptide-Fraction Annotation Differs Strongly Between AM-V and VM-V

Both peptide fractions yield 11 protein groups, but their annotation depth differs markedly (Table 2). AM-V PEP is dominated by melittin precursor and also contains secapin isoform X1, mast cell degranulating peptide preproprotein, allergen Api m 6 precursor, and an omega-conotoxin-like protein entry (Figure 3c; Table 3).

VM-V PEP consists mainly of short transcriptome-derived ORF matches. Stable protein names could not be assigned to the 11 VM-V PEP protein groups, and keyword screening did not recover named wasp venom peptide classes. Thus, the peptide-fraction comparison highlights a strong AM-V peptide annotation signal centered on known honeybee venom peptides and a VM-V peptide fraction that remains largely unannotated at this database resolution. The VM-V PEP data should therefore be interpreted as unresolved short-ORF matches, not as a curated hornet venom peptide catalog.

### 2.4. GO, KEGG, and STRING Profiles Across Protein and Peptide Fractions

**Figure 4:**
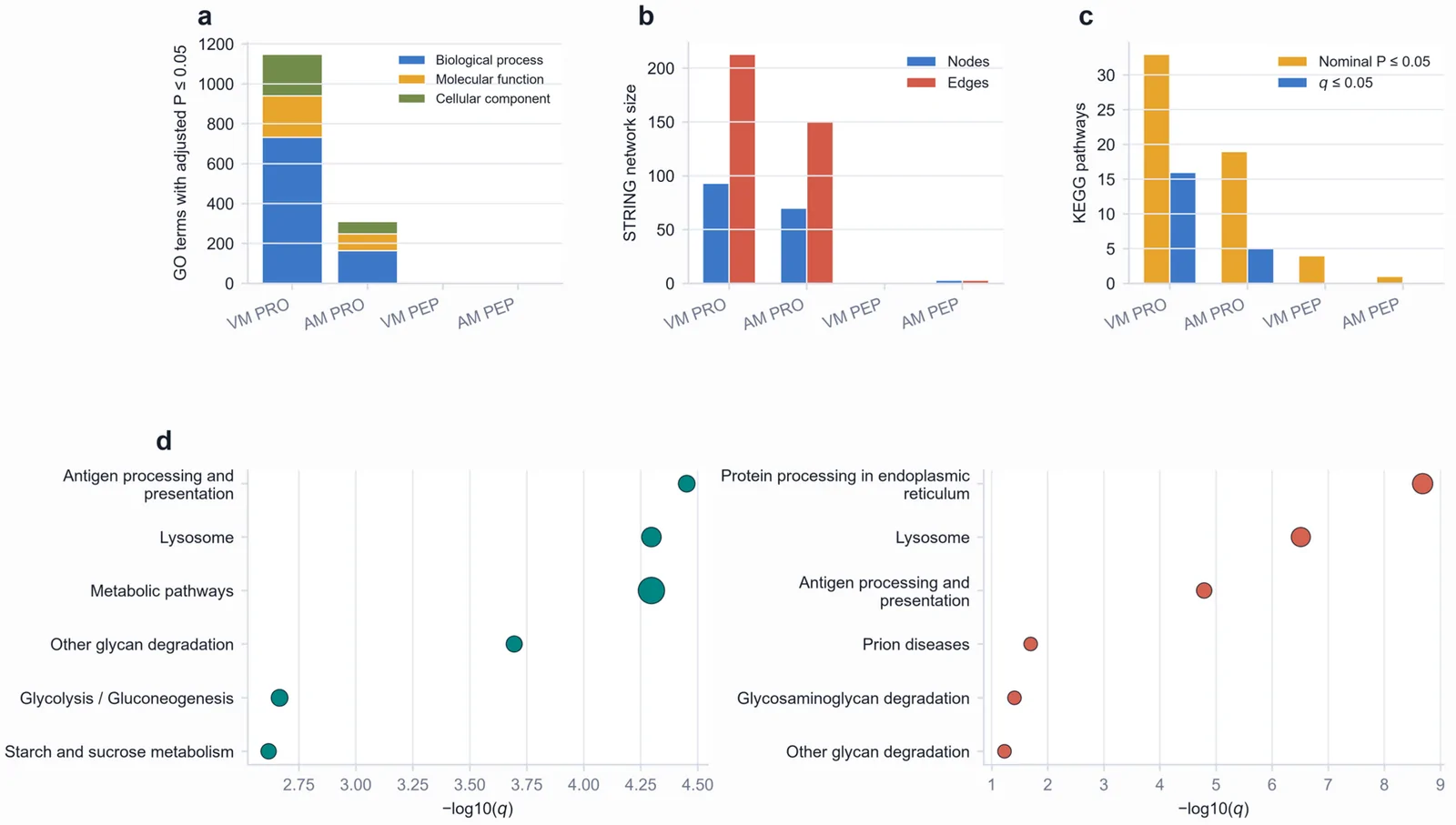
Functional annotation and STRING network summaries. (**a**) GO terms retained at adjusted *p* ≤ 0.05 across biological process, molecular function, and cellular component domains. (**b**) STRING network node and edge counts for protein and peptide fractions. (**c**) KEGG pathways retained at nominal *p* ≤ 0.05 and *q* ≤ 0.05. (**d**) Top KEGG pathways in VM-V PRO and AM-V PRO ranked by *q* value; point size reflects the number of mapped proteins. The outputs are interpreted as functional annotation summaries rather than replicate-level abundance results. Panel-specific legends identify the color assignments for GO terms, STRING nodes and edges, and nominal versus q-significant KEGG pathways. VM-V, *Vespa mandarinia* venom; AM-V, *Apis mellifera* venom; PRO, protein fraction; PEP, peptide fraction; GO, Gene Ontology; KEGG, Kyoto Encyclopedia of Genes and Genomes; STRING, Search Tool for the Retrieval of Interacting Genes/Proteins.

GO enrichment reveals stronger functional annotation structure in the protein fractions than in the peptide fractions (Figure 4a). Because the study was designed as an identification-centered resource rather than a replicate-level quantitative comparison, enrichment results are interpreted as annotation summaries. AM-V PRO contains 62 cellular-component, 84 molecular-function, and 164 biological-process GO terms with adjusted *p* ≤ 0.05. VM-V PRO contains 209 cellular-component, 208 molecular-function, and 732 biological-process GO terms with adjusted *p* ≤ 0.05. AM-V PEP and VM-V PEP produce nominal GO signals without adjusted-significant GO terms. The corresponding GO enrichment summary is provided in Supplementary Table S5.

KEGG enrichment shows a similar protein-level pattern (Figure 4c,d). AM-V PRO contains five pathways with *q* ≤ 0.05, including protein processing in endoplasmic reticulum, lysosome, antigen processing and presentation, prion diseases, and glycosaminoglycan degradation. VM-V PRO contains 16 pathways with *q* ≤ 0.05, including antigen processing and presentation, lysosome, metabolic pathways, other glycan degradation, glycolysis/gluconeogenesis, starch and sucrose metabolism, amino sugar and nucleotide sugar metabolism, glycosaminoglycan degradation, and glycosphingolipid biosynthesis pathways. AM-V PEP contains one nominal KEGG pathway and VM-V PEP contains four nominal KEGG pathways; neither peptide fraction retains *q* ≤ 0.05 KEGG pathways. The corresponding KEGG enrichment summary is provided in Supplementary Table S6.

STRING (v11.0) and Cytoscape (v3.7.2) network outputs further separate the protein and peptide fractions (Figure 4b). AM-V PRO produces a 70-node, 150-edge network with a mean combined score of 0.6503 and a maximum score of 0.999. VM-V PRO produces a 93-node, 213-edge network with a mean combined score of 0.6302 and a maximum score of 0.997. AM-V PEP forms a compact three-node, three-edge network. VM-V PEP has no parsed STRING interaction edges. The network results therefore concentrate the strongest interaction-annotation structure in AM-V PRO and VM-V PRO and are used as hypothesis-generating evidence. The corresponding network summary is provided in Supplementary Table S7.

### 2.5. Transcriptome Resource Supporting VM-V Protein Identification

The de novo transcriptome used to construct the VM-V search database yields 38.63–54.62 million clean reads per sample (mean, 47.29 million), with Q30 values of 92.40–92.79% (mean, 92.56%) (Figure S1). The biological sampling design and database-construction role of these transcriptomes are described in Section 5.5. The transcriptome summary is provided in Supplementary Table S8.

Trinity assembly generates 81,673 transcripts and 35,752 genes. Transcript N50 is 1526 bp, and gene N50 is 1236 bp. Among assembled genes, 11,025 have nonblank annotations, 6844 have assigned names, 7880 have GO-term annotations, 7899 have KO entries, 7899 have KEGG pathway annotations, and 7953 have Pfam descriptions. This transcriptome resource provides the database foundation for VM-V protein identification and supplies functional annotation for the transcriptome-derived VM-V protein entries.

## 3. Discussion

### 3.1. Comparative Proteomic and Peptidomic Features of VM-V and AM-V

This study presents a comparative proteomic and peptidomic identification profile of *V. mandarinia* and *A. mellifera* venoms using paired protein- and peptide-fraction LC-MS/MS datasets. The protein fractions provided the richest identification information, detecting 197 protein groups in VM-V PRO and 164 protein groups in AM-V PRO. The peptide fractions produced smaller datasets, with 11 protein groups in both AM-V PEP and VM-V PEP, and they differed strongly in annotation resolution. These values represent protein-group detection and peptide evidence, not quantitative venom abundance. Together, these datasets describe two venom-sample annotation profiles with distinct levels of database support: a honeybee venom profile anchored by well-curated reference proteins and a hornet venom profile supported by de novo transcriptomic annotation.

AM-V contained the expected suite of honeybee venom-associated components, including phospholipase A2, hyaluronidase, venom acid phosphatase, venom dipeptidyl peptidase 4, venom serine proteases, allergen Api m 6, melittin precursor, mast cell degranulating peptide preproprotein, and secapin. Prior mass-spectrometry work on Africanized and European honeybee venoms resolved venom enzymes and associated post-translational features [8]. A separate honeybee venom proteome study of queens and winter bees further supports the recovery of secreted venom proteins and allergen-related components by proteomic analysis [9]. The peptide-level pattern is also consistent with the established role of melittin in honeybee venom and with analytical work focused on melittin quantification [10]. Api m 6 detection adds allergen-level support because this family is part of the curated honeybee venom allergen repertoire [11]. The strong AM-V peptide annotation, especially the melittin precursor-dominant AM-V PEP identification signal, provides an internal reference point for interpreting the venom fractionation and LC-MS/MS workflow.

VM-V PRO revealed a complementary set of transcriptome-supported candidate proteins. Venom dipeptidyl peptidase 4-like, hyaluronidase-like, venom allergen 5-like, venom serine protease 34-like, and venom serine carboxypeptidase-like entries place VM-V within the broader molecular landscape of Hymenoptera and *Vespa* venoms. Comparative proteomics of *Vespa tropica* and *Vespa affinis* reported hyaluronidase, phospholipase, protease, and antigen 5-like venom components in related *Vespa* species [12]. Proteo-transcriptomic analysis of *V. affinis* supports the use of transcript-derived evidence to interpret hornet venom enzyme and allergen candidates [13]. The recent *V. mandarinia* venom multi-omics study gives species-level context for interpreting these VM-V candidates as part of an emerging venomomics resource for the Asian giant hornet [14]. The detection of apolipophorin-like, hexamerin-like, transferrin-like, lysozyme-like, and related entries further suggests that the VM-V protein fraction may include a broader protein mixture associated with venom collection, secretion, extracellular proteins, and insect hemolymph-linked components. Purified phospholipase, antigen 5, and hyaluronidase components from V. velutina provide additional orthogonal support for these recurring Vespa venom protein families [15].

### 3.2. AM-V Provides a Reference for Interpreting Venom Peptide Annotation

The peptide-fraction comparison highlights the importance of reference database coverage. AM-V PEP showed a clearly interpretable peptide profile, led by melittin precursor and accompanied by secapin, mast cell degranulating peptide preproprotein, allergen Api m 6 precursor, and an omega-conotoxin-like protein entry. These components align with the known honeybee venom peptide repertoire and demonstrate that the peptide-fraction workflow can recover biologically recognizable venom-associated peptides when database annotation is mature [10]. AM-V therefore functions as a workflow and annotation comparator; it is not treated here as a biological control matched to VM-V at the level of donor individuals, colony status, or venom yield.

VM-V PEP presented a different pattern. The 11 VM-V PEP protein groups were short transcriptome-derived ORF matches without stable protein names. This result is informative for *V. mandarinia* venomomics because it separates protein-level candidate discovery from peptide-level annotation maturity. Wasp venoms are known to contain diverse bioactive peptides [16], and the recent comparative wasp peptidomics dataset emphasizes that peptide diversity, proteolytic processing, and predicted bioactivities can vary markedly across wasp venoms [17]. The VM-V PEP dataset therefore provides a starting point for future sequence-centered annotation of short hornet venom peptides.

### 3.3. Functional Annotation Emphasizes Protein-Level Organization

The GO and KEGG results reinforce the protein fractions as the main interpretable layer of the dataset. AM-V PRO and VM-V PRO both retained large numbers of adjusted-significant GO terms, while AM-V PEP and VM-V PEP produced only nominal enrichment signals. KEGG enrichment showed the same pattern, with *q* ≤ 0.05 pathways in AM-V PRO and VM-V PRO and no *q* ≤ 0.05 pathways in the peptide fractions.

The protein-level pathways point to processing, lysosomal, glycan-related, metabolic, and immune-related annotation categories. These categories are compatible with a venom-derived protein mixture containing secreted enzymes, processing-associated proteins, glycoprotein-related enzymes, and broader insect protein components. In VM-V PRO, the prominence of lysosome, glycan degradation, glycolysis/gluconeogenesis, and carbohydrate-related pathways provides a functional annotation framework for organizing transcriptome-derived candidates. In AM-V PRO, protein processing in endoplasmic reticulum, lysosome, antigen processing and presentation, and glycosaminoglycan degradation fit well with a venom proteome containing enzymes, allergens, and secreted proteins. These pathway and network summaries are best interpreted as annotation maps that help prioritize protein families for validation, not as direct evidence of pathway activation or biochemical activity.

STRING/Cytoscape network outputs similarly concentrated the strongest annotation structure in the protein fractions. VM-V PRO produced a larger network than AM-V PRO, with 93 nodes and 213 edges compared with 70 nodes and 150 edges in AM-V PRO. The peptide networks were much smaller, with only three nodes and three edges in AM-V PEP and no parsed interaction edges in VM-V PEP. Because STRING integrates known and predicted protein associations, these network patterns are best used as a guide for choosing representative protein modules and candidate proteins for future targeted validation [18].

### 3.4. Transcriptome Support Expands VM-V Protein Identification

The VM-V dataset illustrates the value of transcriptome-supported proteomics in a non-model venomous insect. The de novo transcriptome provided 81,673 transcripts and 35,752 genes, with more than 11,000 annotated genes and nearly 7900 genes linked to GO or KEGG annotations. This resource enabled the construction of a VM-V protein search database and allowed many VM-V protein groups to receive functional names through transcriptome-supported annotation. The strategy matches the broader proteo-transcriptomic logic used for hornet venom interpretation, where venom-gland or species-specific transcript resources improve protein naming and candidate prioritization [13].

The resulting identifications remain conditional on the sequence database. Incomplete transcripts and partial open reading frames can produce truncated protein models, while overlapping or redundant isoforms can distribute peptide evidence among near-identical entries and complicate protein-group assignment. Homology-based annotation can also propagate uncertain functional names, and differences in database completeness create unequal opportunities for identification in VM-V and the more mature *A. mellifera* reference resource [19]. These effects are particularly relevant to the unresolved short-ORF matches in VM-V PEP. Accordingly, the numbers and functional labels recovered from the two search spaces should not be interpreted as direct measures of species-level venom complexity.

The availability of a chromosome-level *V. mandarinia* genome now creates a clear path for further improvement. Reannotation of VM-V spectra against updated genome-derived protein models could improve protein naming, reduce transcript-derived redundancy, and refine the relationship between VM-V protein candidates and genomic loci. The present transcriptome-supported dataset therefore serves both as an interpretable venom proteomic resource and as a baseline for future genome-informed reanalysis.

### 3.5. Relationship to Recent Vespa Venom Research

Recent hornet venomics studies demonstrate how analytical design and reference resources determine the level of biological inference. Quantitative SWATH-MS studies of *V. velutina nigrithorax* venom sacs [20] and *V. crabro* venom-sac extracts [21] resolved caste-associated protein patterns using replicated within-species designs. These studies provide quantitative biological comparisons that complement, but are methodologically distinct from, the identification-level comparison of commercial venom materials presented here. Integrated profiling of *A. dorsata* and *Vespa* sp. venoms recently recovered established bee venom components together with homology-supported Vespa phospholipase-, DPP4-, serpin-, PNGase-, and QPCT-like candidates [22]. That work independently supports comparative, database-assisted candidate screening, while its metabolomic, docking, and bioactivity-prioritization analyses address questions beyond the present dataset. Species-level multi-omics of *V. mandarinia* [14], comparative wasp peptidomics [17], and toxicological analysis of *V. mandarinia* venom exposure [23] further frame the present dataset as a complementary identification and annotation resource.

This comparative design strengthens interpretation by anchoring the analysis in a mature honeybee venom model and extending the same workflow to a less curated hornet venom dataset. The main scientific value of the present work lies in the organized comparison of protein-level candidates, peptide-level annotation contrast, and functional annotation structure across VM-V and AM-V.

### 3.6. Limitations

Several limitations should be considered when interpreting the results. VM-V and AM-V were analyzed as commercially sourced venom materials without independent biological replication; therefore, the comparison is restricted to the protein and peptide repertoires identified in the analyzed materials and does not establish population-level or species-level differences across donor individuals, colonies, collection dates, developmental states, or geographic origins. Species assignment followed the supplier documentation. The three LC-MS/MS injections per sample provided technical identification support but did not substitute for biological replication. The study design therefore supports identification, annotation, and candidate prioritization, but not strict differential-abundance testing or inferential comparison between species.

The transcriptome dataset supports VM-V database construction and consists of venom-sac samples from five Yunnan production origins purchased from the same breeding company. In this study, Y1–Y5 are used to support database construction and annotation rather than to infer origin-specific venom-sac expression differences. GO, KEGG, and STRING analyses provide annotation organization and candidate prioritization, with additional uncertainty introduced by predefined protein sets. The candidate VM-V proteins were not independently confirmed by targeted mass spectrometry, immunochemical detection, recombinant expression, or activity assays. Experimental activity, allergenicity, ecological function, pharmacological potential, and definitive toxin status therefore require future validation.

## 4. Conclusions

This study provides an identification-based comparative proteomic and peptidomic resource for the analyzed *V. mandarinia* and *A. mellifera* venom materials. The AM-V dataset recovered established honeybee venom proteins and peptides, including phospholipase A2, hyaluronidase, venom acid phosphatase, venom dipeptidyl peptidase 4, melittin precursor, secapin, mast cell degranulating peptide precursor, and allergen Api m 6. The VM-V dataset yielded a transcriptome-supported candidate set that included venom dipeptidyl peptidase 4-like, hyaluronidase-like, venom allergen 5-like, serine protease-like, apolipophorin-like, and hexamerin-like entries. Functional annotation and STRING networks organized the protein-level identifications and provided a basis for candidate prioritization. These identification data support targeted mass-spectrometric or orthogonal validation and genome-informed reanalysis of candidate *V. mandarinia* venom-associated components; they do not establish quantitative species-level differences or biological activity.

## 5. Materials and Methods

### 5.1. Venom Samples and Fractionation

Two commercial crude venom materials were used for proteomic and peptidomic analysis. VM-V was *V. mandarinia* venom purchased from Dehong Hufeng Breeding Co., Ltd. (Dehong, Yunnan, China), and AM-V was Italian honeybee (*A. mellifera*) venom purchased from Anhui Jinfeng Bee Venom Biotechnology Development Co., Ltd. (Maanshan, Anhui, China). Both preparations had been collected from live insects by electrical stimulation; dissected venom sacs were not used for the proteomic or peptidomic analyses. Species assignments followed the supplier records. The samples were transported on dry ice, stored at −80 °C, and assigned the identifiers P19060-VM-V and P19060-AM-V. Each venom material was separated by ultrafiltration into protein and peptide fractions, generating VM-V PRO, AM-V PRO, VM-V PEP, and AM-V PEP.

A separate set of 15 *V. mandarinia* venom-sac samples was used only for de novo transcriptome assembly and construction of the VM-V protein search database (Section 5.5). These venom sacs were not pooled with the commercial VM-V material and were not analyzed in the proteomic or peptidomic workflow.

Frozen venom samples were dissolved in 25 mmol/L NH_4_HCO_3_ and concentrated at 4 °C and 13,000× *g* using VIVACON 500 centrifugal filter units with a 10 kDa molecular-weight cutoff (Sartorius, Göttingen, Germany; catalog no. VN01H02). The concentrate was washed three times with 25 mmol/L NH_4_HCO_3_, reverse-spun, and mixed with an equal volume of SDT lysis buffer containing 4% SDS, 100 mmol/L Tris-HCl, and 100 mmol/L DTT at pH 8.0. The mixture was heated at 100 °C for 10 min and centrifuged at 13,000× *g* for 15 min. The supernatant was used for BCA protein quantification and SDS-PAGE. The peptide fraction obtained by ultrafiltration was combined and concentrated under low-temperature vacuum.

### 5.2. SDS-PAGE and FASP Digestion

For SDS-PAGE, 20 μg of each venom protein sample was mixed with 2 μL of 5× loading buffer containing 250 mmol/L Tris-HCl (pH 6.8), 10% (*w*/*v*) SDS, 0.5% (*w*/*v*) bromophenol blue, 50% (*v*/*v*) glycerol, and 5% DTT. Samples were heated at 98 °C for 3 min, cooled to room temperature, and centrifuged at 3000× *g* for 1 min before electrophoresis. A 1× running buffer containing 25 mmol/L Tris, 192 mmol/L glycine, and 0.1% (*w*/*v*) SDS was prepared by diluting a 10× stock (30.3 g Tris base, 144.0 g glycine, and 10.0 g SDS brought to 1 L with ultrapure water; no pH adjustment; final pH approximately 8.3) 1:10 with ultrapure water. Electrophoresis was performed at 100 V for 10 min followed by 200 V for 30 min. Gels were rinsed with deionized water, stained using a Coomassie Brilliant Blue R-250 staining solutions kit (Bio-Rad Laboratories, Hercules, CA, USA; catalog no. 1610435), and scanned. SDS-PAGE was used solely for visual quality control of the bulk protein preparations. No gel bands were excised or subjected to in-gel digestion.

FASP was performed independently on aliquots of the bulk liquid protein concentrates rather than on excised gel material. Briefly, 100 μg of each protein sample was reduced with DTT, treated with UA buffer containing 8 mol/L urea and 150 mmol/L Tris-HCl at pH 8.5, alkylated with 50 mmol/L IAA, washed with UA buffer and ammonium bicarbonate buffer, and digested with trypsin at 37 °C for 18 h. Digested peptides were collected by centrifugation, quantified by OD280, and used for LC-MS/MS analysis.

### 5.3. LC-MS/MS Analysis

Peptides were separated using an EASY-nLC 1200 system (Thermo Fisher Scientific/Proxeon Biosystems A/S, Odense, Denmark) and analyzed on an Orbitrap Fusion Lumos mass spectrometer (Thermo Fisher Scientific, San Jose, CA, USA). Solvent A was 0.1% formic acid in water, and solvent B was 0.1% formic acid in acetonitrile. Samples were loaded onto an EASY trap column and separated on a Thermo Scientific C_18_ analytical column (Thermo Fisher Scientific, Waltham, MA, USA) (75 μm × 25 cm, 5 μm, 100 Å). The 60 min gradient increased solvent B from 5% to 28% over 40 min, from 28% to 90% over 2 min, and then maintained 90% B until 60 min.

MS was performed in positive-ion mode. The precursor scan range was 375–1800 *m*/*z*. MS_1_ resolution was 120,000 at *m*/*z* 200, with an AGC target of 4 × 10^5^ and maximum injection time of 50 ms. Data-dependent acquisition was performed in top-speed mode with a cycle time of 3 s and dynamic exclusion of 40 s. MS_2_ spectra were acquired using HCD at a resolution of 50,000 at *m*/*z* 200, with an AGC target of 1 × 10^5^ and maximum injection time of 105 ms. Each sample type was analyzed in three LC-MS/MS injections, which were treated as technical identification support rather than independent biological replicates.

### 5.4. Database Searching and Protein Identification

Raw MS files were searched using MaxQuant 1.5.3.0 [24]. VM-V PRO was searched against Trinity.gene.fasta.transdecoder.pep.fasta, AM-V PRO against GCF_003254395.2_Amel_HAv3.1_protein.fasta, VM-V PEP against P16060-15K.fasta, and AM-V PEP against GCF_003254395.2_Amel_HAv3.1_15K.fasta. Variable modifications were oxidation (M) and acetylation (protein N-term). Carbamidomethylation (C) was used as a fixed modification for protein samples; the fixed modification field was blank for peptide samples. Digestion mode was Trypsin/P for protein samples and unspecific for peptide samples. Up to two missed cleavages were allowed. Main search tolerance was 6 ppm, FTMS MS/MS tolerance was 20 ppm, and the minimum peptide length was two amino acids. Decoy mode was set to Revert. PSM FDR and protein FDR were both set to 0.01. Label-free quantification and iBAQ were enabled, and Unique + Razor peptides were used for quantification. The downstream analysis used protein-group and peptide-identification evidence to support candidate annotation; replicate-level LFQ/iBAQ abundance statistics were not performed.

### 5.5. Transcriptome Assembly and Annotation

The VM-V search database was constructed from a de novo *V. mandarinia* venom-sac transcriptome. Fifteen venom-sac samples represented five Yunnan production-origin groups (Y1–Y5), with three biological samples per group; all were purchased from the same breeding company. The Y1–Y5 materials were used for database construction and annotation, not for origin-specific expression analysis. Reads were assembled with Trinity [25]. De novo assembly and annotation can expand the searchable protein space for non-model organisms while introducing assembly, ORF-prediction, and annotation uncertainties that require conservative interpretation [19]. Transcriptome quality and annotation summaries included clean-read statistics, N50 metrics, gene and transcript counts, gene names, GO terms, KO entries, KEGG pathway annotations, and Pfam descriptions.

### 5.6. GO, KEGG, and STRING Analyses

GO and KEGG enrichment analyses were performed for AM-V PRO, VM-V PRO, AM-V PEP, and VM-V PEP. The enrichment workflow used predefined protein sets from the proteomic analysis as target sets and all identified proteins as the background. Hypergeometric testing was used. Nominal *p* values and adjusted *p*/*q* values were used to summarize enriched terms, with adjusted *p* ≤ 0.05 for GO and *q* ≤ 0.05 for KEGG used to define retained enrichment. GO terms were interpreted as structured functional annotations, not as experimental activity measurements [26]. STRING v11.0 interaction outputs and Cytoscape v3.7.2 node/edge exports were parsed to summarize network nodes, edges, and combined scores for each group [18].

### 5.7. Candidate Component Screening and Data Summarization

Representative venom-associated candidates were screened from protein-identification results using keyword categories relevant to Hymenoptera venom proteins and peptides, including phospholipase, hyaluronidase, venom dipeptidyl peptidase, acid phosphatase, serine protease, carboxylesterase, melittin, secapin, allergen, mast cell degranulating peptide, apolipophorin, hexamerin, transferrin, lysozyme, and venom-named entries. AM-V candidate screening used reference FASTA headers; VM-V screening used transcriptome-supported protein-name annotations. Keyword screening was used as a transparent candidate-prioritization method, not as a toxin-domain classifier, and may have missed unannotated or novel venom peptides. Summary tables were compiled from the protein-identification, peptide-identification, FASTA, GO, KEGG, and STRING results.

## Figures and Tables

**Table 1:** Sample processing, LC-MS/MS datasets, and database-search overview. VM-V, *Vespa mandarinia* venom; AM-V, *Apis mellifera* venom; PRO, protein fraction; PEP, peptide fraction; LC-MS/MS, liquid chromatography-tandem mass spectrometry.

Dataset	Sample Material	Fraction	Sample Processing/Amount	Search Database	LC-MS/MS Replicates
VM-V PRO	*Vespa mandarinia* venom, P19060-VM-V	Protein fraction	Protein concentration 22.99 μg/μL; 200 μL; total 4598 μg	Trinity.gene.fasta.transdecoder.pep.fasta	3
AM-V PRO	*Apis mellifera* venom, P19060-AM-V	Protein fraction	Protein concentration 12.86 μg/μL; 200 μL; total 2572 μg	GCF_003254395.2_Amel_HAv3.1_protein.fasta	3
VM-V PEP	*Vespa mandarinia* venom, P19060-VM-V	Peptide fraction	Peptide amount 2.413 μg/μL; 100 μL; total 241.3 μg	P16060-15K.fasta	3
AM-V PEP	*Apis mellifera* venom, P19060-AM-V	Peptide fraction	Peptide amount 1.935 μg/μL; 100 μL; total 193.5 μg	GCF_003254395.2_Amel_HAv3.1_15K.fasta	3

Ultrafiltration yielded protein and peptide fractions from both venom materials. Sample quantities, LC-MS/MS injections, and the databases used for each dataset are reported in Table 1.

**Table 2:** Protein and peptide identification statistics. VM-V, *Vespa mandarinia* venom; AM-V, *Apis mellifera* venom; PRO, protein fraction; PEP, peptide fraction.

Dataset	Protein Groups	Peptide Rows	Total Peptide Count in Protein Table	Unique Peptide Count Sum	Median Peptides per Protein Group	Maximum Peptides in One Entry	Named Entries
VM-V PRO	197	838	842	834	2	65	171/197 transcriptome-supported named entries
AM-V PRO	164	747	775	714	2	37	Annotated by reference FASTA headers
VM-V PEP	11	19	19	19	2	3	0/11 transcriptome-supported named entries
AM-V PEP	11	58	58	58	1	30	Annotated by reference FASTA headers

**Table 3:** Representative venom-associated candidate proteins and peptides. VM-V, *Vespa mandarinia* venom; AM-V, *Apis mellifera* venom; PRO, protein fraction; PEP, peptide fraction.

Dataset	Representative Entry	Peptides	Candidate Category	Interpretation in Manuscript
VM-V PRO	Apolipophorin-like	65	Apolipophorin-like protein	High-peptide-count transcriptome-supported VM-V protein entry
VM-V PRO	Venom dipeptidyl peptidase 4-like	28	Venom enzyme-like protein	Candidate venom-associated enzyme
VM-V PRO	Hexamerin-like	27	Storage/hemolymph-linked protein	Detected VM-V protein candidate
VM-V PRO	Hyaluronidase-like	17	Venom enzyme-like protein	Candidate spreading factor/venom-associated enzyme family
VM-V PRO	Transferrin-like	14	Transport/immune-related protein	Detected transcriptome-supported protein
VM-V PRO	Venom allergen 5-like	8	Allergen-like venom protein	Candidate allergen-like venom-associated protein
AM-V PRO	Apolipophorin	37	Apolipophorin	High-peptide-count AM-V protein entry
AM-V PRO	Hyaluronidase isoform X1	28	Venom enzyme/allergen family	Known honeybee venom-related protein family
AM-V PRO	Venom dipeptidyl peptidase 4	28	Venom enzyme	Known honeybee venom-related protein
AM-V PRO	Venom acid phosphatase Acph-1 isoforms	20	Venom enzyme	Known honeybee venom-related protein
AM-V PRO	Phospholipase A2 precursor	17	Major honeybee venom enzyme	Established honeybee venom component
AM-V PEP	Melittin precursor	30	Honeybee venom peptide precursor	Dominant AM-V PEP annotation signal
AM-V PEP	Secapin isoform X1	6	Honeybee venom peptide precursor	AM-V peptide-fraction component
AM-V PEP	Mast cell degranulating peptide preproprotein	1	Honeybee venom peptide precursor	AM-V peptide-fraction component
AM-V PEP	Allergen Api m 6 precursor	1	Honeybee venom allergen	AM-V peptide-fraction component

## Data Availability

The data supporting this study are provided in the manuscript, figures, and Supplementary Tables. The Supplementary Tables workbook includes protein and peptide identification summaries, candidate screening tables, GO/KEGG/STRING summaries, transcriptome summary metrics, sample quantification records, and FASTA record summaries. Additional processed data can be made available from the corresponding author upon reasonable request, subject to applicable institutional permissions.

## Supplementary Materials

The following supporting information can be downloaded at https://www.mdpi.com/article/10.3390/toxins18080351/s1: Figure S1, transcriptome sequencing, assembly, and annotation summary supporting VM-V database construction; Table S1, protein-identification summary; Table S2, peptide-identification row counts; Table S3, venom-associated candidate component screening; Table S4, candidate component summary by dataset; Table S5, GO enrichment summaries and representative terms; Table S6, KEGG enrichment summaries and representative pathways; Table S7, STRING/Cytoscape network summaries and representative edges; Table S8, transcriptome sequencing, assembly, and annotation summary; and additional supporting tables for sample quantification records and FASTA record summary.
