# Supplementary material for: Identification-Based Comparative Proteomic and Peptidomic Profiling of Vespa mandarinia and Apis mellifera Venoms Supported by De Novo Transcriptomic Annotation
Source: Toxins (Basel). 2026 Aug 17;18(8):351. doi: 10.3390/toxins18080351 (PMC13517962; doi:10.3390/toxins18080351)
Supplement: Supplementary file 1 [file toxins-18-00351-s001.zip › Supplementary_Figure_S1_transcriptome sequencing, assembly, and annotation summary supporting VM-V database construction.pdf]

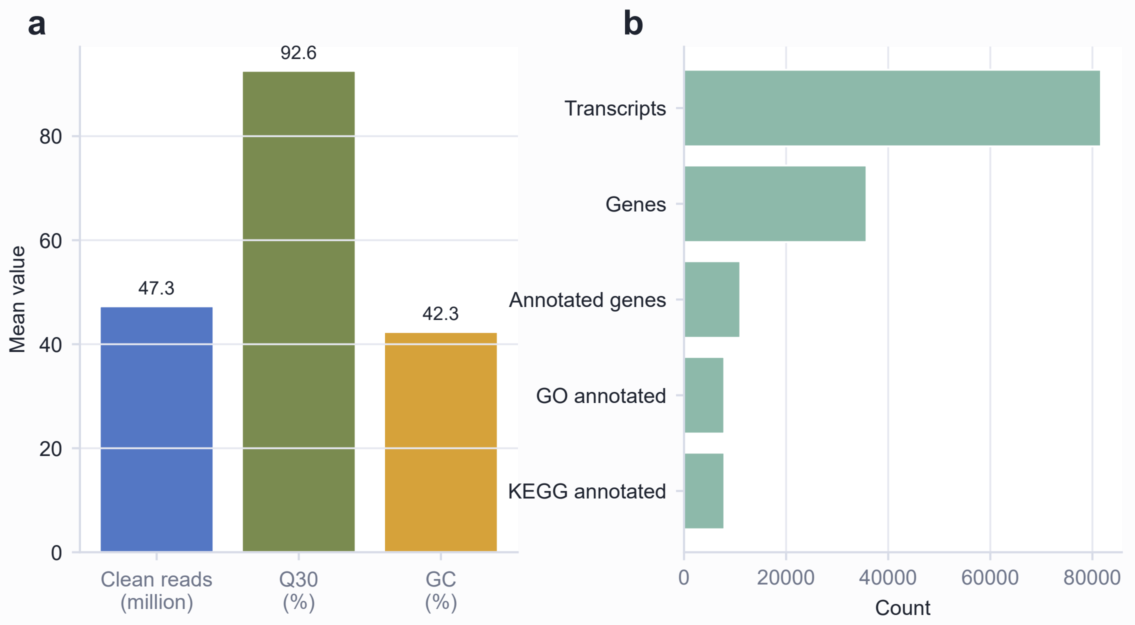

a
Mean value
0
20
40
60
80
47.3
92.6
42.3
Clean reads (million)
Q30 (%)
GC (%)
b
Transcripts
Genes
Annotated genes
GO annotated
KEGG annotated
0
20000
40000
60000
80000
Count
