# Supplementary material for: Identification-Based Comparative Proteomic and Peptidomic Profiling of Vespa mandarinia and Apis mellifera Venoms Supported by De Novo Transcriptomic Annotation
Source: Toxins (Basel). 2026 Aug 17;18(8):351. doi: 10.3390/toxins18080351 (PMC13517962; doi:10.3390/toxins18080351)
Supplement: Supplementary file 1 [file toxins-18-00351-s001.zip › Supplementary_Tables_S1-S8.pdf]

Supplementary Tables S1-S8

This Word file contains the supplementary tables cited in the manuscript. Long cell contents are shortened for Word readability; the accompanying Excel workbook contains the complete structured tables.

Table S1. Protein-identification summary

S1_protein_summary

dataset	protein_groups	peptide_count_sum	peptide_count_median	peptide_count_max	unique_peptide_sum	sequence_coverage_median	sequence_coverage_max	mw_min_kda	mw_median_kda	mw_max_kda	named_entries
AM-V PEP	11	58	1	30	58	9.8	44.3	5.7671	9.8188	12.299	
AM-V PRO	164	775	2	37	714	8.3	82	5.2233	44.835	499.49	
VM-V PEP	11	19	2	3	19	8.8	15.7	10.401	11.714	12.353	
VM-V PRO	197	842	2	65	834	8.5	70.5	11.381	37.881	375.18	
VM-V PRO named	197	842	2	65	834	8.5	70.5	11.381	37.881	375.18	171/197
VM-V PEP named	11	19	2	3	19	8.8	15.7	10.401	11.714	12.353	0/11
Pupa named	1821	5770	2	86	5697	5.9	57.9	11.107	44.675	884.09	1699/1821

Table S2. Peptide-identification row counts

S2_peptide_row_counts

dataset	peptide_rows
AM-V PEP peptides	58
AM-V PRO peptides	747
VM-V PEP peptides	19
VM-V PRO peptides	838

Table S3. Venom-associated candidate component screening

S3_candidate_components

dataset	protein_id	accession	name_or_header	peptides	unique_peptides	sequence_coverage_percent	mw_kda	keyword_categories
AM-VPRO	NP_001011614.1	NP_001011614.1	NP_001011614.1 phospholipase A2 precursor [Apis mellifera]	17	17	82	19.057	phospholipase
AM-VPRO	XP_016767538.1	XP_016767538.1;XP_016767537.1;XP_016767536.1;XP_016767535.1;XP_006569952.1;NP_001013377.2	XP_016767538.1 venom acid phosphatase Acph-1 isoform X1 [Apis mellifera]; XP_016767537.1 venom acid phosphatase Acph-1 isoform X1 [Apis mellifera]; XP_016767536.1 venom acid phosp...	20	20	54.1	46.871	venom acid phosphatase; venom-named
AM-VPRO	XP_026300654.1	XP_026300654.1	XP_026300654.1 hyaluronidase isoform X1 [Apis mellifera]	28	3	33.2	84.684	hyaluronidase
AM-VPR	NP_001035360.1	NP_001035360.1	NP_001035360.1 allergen Api m 6 precursor	4	4	34.8	9.8188	allergen

O			[Apis mellifera]					
AM-VPRO	XP_026299326.1	XP_026299326.1;NP_001119715.2	XP_026299326.1 venom dipeptidyl peptidase 4 isoform X1 [Apis mellifera]; NP_001119715.2 venom dipeptidyl peptidase 4 precursor [Apis mellifera]	28	28	44	88.009	venom dipeptidyl peptidase; venom-named
AM-VPRO	XP_001120293.2	XP_001120293.2	XP_001120293.2 phospholipase A2-like [Apis mellifera]	7	7	41.6	20.447	phospholipase
AM-VPRO	NP_001119716.1	NP_001119716.1	NP_001119716.1 venom carboxylesterase-6 precursor [Apis mellifera]	14	14	27.1	63.636	carboxylesterase; venom-named
AM-VPRO	XP_026300310.1	XP_026300310.1;NP_001011584.1	XP_026300310.1 venom serine protease 34 isoform X1 [Apis mellifera]; NP_001011584.1 venom serine protease 34 precursor [Apis mellifera]	14	14	46.7	42.137	serine protease; venom-named

AM-VPRO	XP_016770111.1	XP_016770111.1	XP_016770111.1 nucleobindin-2 [Apis mellifera]	16	16	27.3	63.299	nucleobindin
AM-VPRO	XP_392381.3	XP_392381.3;XP_006567715.1	XP_392381.3 putative cysteine proteinase CG12163 isoform X2 [Apis mellifera]; XP_006567715.1 putative cysteine proteinase CG12163 isoform X1 [Apis mellifera]	19	19	25.9	100.38	cysteine proteinase
AM-VPRO	NP_001011619.1	NP_001011619.1	NP_001011619.1 hyaluronidase precursor [Apis mellifera]	27	2	62	44.259	hyaluronidase
AM-VPRO	XP_016771167.1	XP_016771167.1;NP_001152775.1	XP_016771167.1 venom serine carboxypeptidase isoform X1 [Apis mellifera]; NP_001152775.1 venom serine carboxypeptidase precursor [Apis mellifera]	5	5	14.1	53.719	venom-named
A	XP_026300665.1	XP_026300665.1;NP_001138311.1	XP_02630	4	4	22.7	17.	venom-

M-VPRO			0665.1 C1q-like venom protein isoform X1 [Apis mellifera]; NP_001138311.1 C1q-like venom protein precursor [Apis mellifera]				042	named
AM-VPRO	XP_026300525.1	XP_026300525.1	XP_026300525.1 lysozyme-like [Apis mellifera]	6	6	42.3	17.835	lysozyme
AM-VPRO	XP_026296694.1	XP_026296694.1;XP_006569959.1	XP_026296694.1 venom acid phosphatase Acph-1 [Apis mellifera]; XP_006569959.1 venom acid phosphatase Acph-1 [Apis mellifera]	7	7	22.7	42.665	venom acid phosphatase; venom-named
AM-VPRO	XP_006560620.1	XP_006560620.1	XP_006560620.1 venom serine protease Bi-VSP [Apis mellifera]	7	7	23.5	39.483	serine protease; venom-named
AM-VPR	XP_026298285.1	XP_026298285.1	XP_026298285.1 apolipophorins [Apis	37	37	12.4	376.8	apolipophorin

O			mellifera]					
AM-VPRO	XP_026296056.1	XP_026296056.1;XP_392669.1	XP_026296056.1 venom serine protease 34 isoform X2 [Apis mellifera]; XP_392669.1 venom serine protease 34 isoform X1 [Apis mellifera]	5	5	16.9	44.438	serine protease; venom-named
AM-VPRO	NP_001011572.1	NP_001011572.1	NP_001011572.1 transferrin 1 precursor [Apis mellifera]	12	12	19.5	78.657	transferrin
AM-VPRO	NP_001104234.1	NP_001104234.1	NP_001104234.1 hexamerin 70a precursor [Apis mellifera]	5	5	7.7	81.459	hexamerin
AM-VPRO	XP_026300858.1	XP_026300858.1;XP_016773435.2;XP_001122067.3	XP_026300858.1 serine protease inhibitor 27A isoform X3 [Apis mellifera]; XP_016773435.2 serine protease inhibitor 27A isoform X2 [Apis mellifera]; XP_001122067.3	5	5	12.5	47.075	serine protease

			serine protease ...					
AM-VPRO	NP_001011607.1	NP_001011607.1	NP_001011607.1 melittin precursor [Apis mellifera]	1	1	20	7.5848	melittin
AM-VPRO	NP_001011611.2	NP_001011611.2	NP_001011611.2 mast cell degranulating peptide preproprotein [Apis mellifera]	1	1	18	5.7671	mast cell degranulating peptide
AM-VPRO	XP_016767767.2	XP_016767767.2;NP_001011618.1	XP_016767767.2 secapin isoform X1 [Apis mellifera]; NP_001011618.1 secapin preproprotein [Apis mellifera]	1	1	10.4	8.6643	secapin
AM-VPRO	NP_001107670.1	NP_001107670.1	NP_001107670.1 apolipophorin-III-like protein precursor [Apis mellifera]	4	4	37.3	21.348	apolipophorin
AM-VPRO	XP_001120594.2	XP_001120594.2	XP_001120594.2 modular serine protease [Apis mellifera]	2	2	3.5	73.331	serine protease
AM-VPR	XP_006560077.1	XP_006560077.1	XP_006560077.1 omega-conotoxin-like	2	2	41.9	8.2976	omega-conotoxin-like

O			protein 1 [Apis mellifera]					
AM-VPRO	XP_006562600.2	XP_006562600.2	XP_006562600.2 digestive cysteine proteinase 1 [Apis mellifera]	2	2	3.1	62.621	cysteine proteinase
AM-VPRO	XP_026302056.1	XP_026302056.1;XP_016771626.2;XP_016771623.2;XP_016771619.2;XP_016771610.2;XP_016771585.2;XP_006562832.2;XP_006562831.2;XP_006562830.2;XP_006562829.2;XP_006562828.2;XP_006562827.2;XP_006562833.2	XP_026302056.1 venom metalloproteinase 3 isoform X4 [Apis mellifera]; XP_016771626.2 A disintegrin and metalloproteinase with thrombospondin motifs 8 isoform X3 [Apis mellifera]; ...	6	6	11.2	66.619	venom-named
AM-VPRO	XP_006563418.1	XP_006563418.1;XP_006563415.1	XP_006563418.1 cysteine-rich venom protein 6 isoform X2 [Apis mellifera]; XP_006563415.1 cysteine-rich venom protein 1 isoform X1 [Apis mellifera]	1	1	19.8	10.191	venom-named

AM-VPRO	XP_026297212.1	XP_026297212.1;XP_006570612.1;XP_006570611.1;XP_006570610.1;XP_016768147.1	XP_026297212.1 glucosylceramidase-like isoform X2 [Apis mellifera]; XP_006570612.1 glucosylceramidase-like isoform X2 [Apis mellifera]; XP_006570611.1 glucosylceramidase-like isof...	3	3	3.5	57.952	glucosylceramidase
AM-VPRO	XP_006570673.1	XP_006570673.1;XP_624472.3	XP_006570673.1 group XV phospholipase A2 isoform X2 [Apis mellifera]; XP_624472.3 group XV phospholipase A2 isoform X1 [Apis mellifera]	1	1	2.9	43.371	phospholipase
AM-VPRO	XP_016772088.2	XP_016772088.2	XP_016772088.2 serine protease snake [Apis mellifera]	1	1	2.7	50.206	serine protease
AM-VPEP	NP_001011607.1	NP_001011607.1	NP_001011607.1 melittin precursor [Apis mellifera]	30	30	44.3	7.5848	melittin

AM-VPEP	XP_016767767.2	XP_016767767.2	XP_016767767.2 secapin isoform X1 [Apis mellifera]	6	6	24.7	8.6643	secapin
AM-VPEP	XP_006560077.1	XP_006560077.1	XP_006560077.1 omega-conotoxin-like protein 1 [Apis mellifera]	4	4	16.2	8.2976	omega-conotoxin-like
AM-VPEP	NP_001011611.2	NP_001011611.2	NP_001011611.2 mast cell degranulating peptide preproprotein [Apis mellifera]	1	1	18	5.7671	mast cell degranulating peptide
AM-VPEP	NP_001035360.1	NP_001035360.1	NP_001035360.1 allergen Api m 6 precursor [Apis mellifera]	1	1	9.8	9.8188	allergen
VM-VPRO	Gene.1004::TRINITY_DN12660_c0_g2::g.1004::m.1004	XP_003487472.1	Lysozyme 2-like	2	2	12.6	16.471	lysozyme
VM-VPRO	Gene.11325::TRINITY_DN14842_c2_g3::g.11325::m.11325	XP_003484995.1	Hyaluronidase-like	13	13	48.7	27.644	hyaluronidase
VM-VPRO	Gene.1328::TRINITY_DN12729_c0_g1::g.1328::m.1328	XP_003494658.1	Venom serine carboxypeptidase-like	2	2	5.1	53.838	venom-named
VM	Gene.13352::TRINITY_DN15255_c0_g2::g.13	XP_003486912.1	Transferrin-like	14	14	26.8	67.74	transferrin

-V<br>P<br>R<br>O	352::m.13352						1	
V<br>M<br>-V<br>P<br>R<br>O	Gene.15333::TRINITY_DN15642_c1_g1::g.15333::m.15333	XP_003492869.1	Hexamerin-like	27	27	50.9	78.557	hexamerin
V<br>M<br>-V<br>P<br>R<br>O	Gene.15383::TRINITY_DN15652_c2_g4::g.15383::m.15383	XP_003491784.1	Apolipophorins-like	65	65	22.3	375.18	apolipophorin
V<br>M<br>-V<br>P<br>R<br>O	Gene.1655::TRINITY_DN12800_c1_g14::g.1655::m.1655	XP_003491166.1	Trehalase-like	12	12	24.3	69.25	trehalase
V<br>M<br>-V<br>P<br>R<br>O	Gene.16561::TRINITY_DN15944_c0_g1::g.16561::m.16561	XP_003487633.1	Lysozyme c-1-like	3	3	19.3	17.249	lysozyme
V<br>M<br>-V<br>P<br>R<br>O	Gene.1694::TRINITY_DN12808_c1_g3::g.1694::m.1694	XP_003487239.1	Venom dipeptidyl peptidase 4-like	28	28	48.5	88.522	venom dipeptidyl peptidase; venom-named
V<br>M<br>-V<br>P<br>R<br>O	Gene.17472::TRINITY_DN9903_c0_g1::g.17472::m.17472	XP_003493883.1	Venom allergen 5-like	8	8	33.8	25.163	allergen; venom-named
V<br>M<br>-V<br>P<br>R<br>O	Gene.2374::TRINITY_DN12965_c2_g1::g.2374::m.2374	XP_003492870.1	Hexamerin-like	23	23	49.7	61.389	hexamerin
V<br>M<br>-V	Gene.3905::TRINITY_DN13263_c1_g7::g.3905::m.3905	XP_003488253.1	Hexamerin-like	5	5	23.3	47.607	hexamerin

PRO								
VM-VPRO	Gene.4222::TRINITY_DN13334_c1_g15::g.4222::m.4222	XP_003491134.1	Glucosylceramidase-like	11	11	20.3	59.548	glucosylceramidase
VM-VPRO	Gene.5616::TRINITY_DN13637_c1_g6::g.5616::m.5616	XP_003484686.1	Nucleobindin-2-like	15	15	34.2	60.295	nucleobindin
VM-VPRO	Gene.7375::TRINITY_DN14006_c2_g5::g.7375::m.7375	XP_003484995.1	Hyaluronidase-like	17	17	57.3	32.855	hyaluronidase
VM-VPRO	Gene.8458::TRINITY_DN14240_c0_g2::g.8458::m.8458	XP_003486731.1	Venom serine protease 34-like	2	2	6.2	44.85	serine protease; venom-named
VM-VPRO	Gene.939::TRINITY_DN12648_c1_g1::g.939::m.939	XP_003492862.1	Hexamerin-like	16	16	27.7	81.658	hexamerin
VM-VPRO	Gene.9909::TRINITY_DN14562_c0_g3::g.9909::m.9909	XP_003488253.1	Hexamerin-like	3	3	19.5	18.067	hexamerin

Table S4. Candidate component summary by dataset

S4_candidate_summary

dataset	total_protein_groups	keyword_matched_candidates	keyword_class_counts
AM-V PEP	11	5	allergen:1; mast cell degranulating peptide:1; melittin:1; omega-conotoxin-like:1; secapin:1
AM-V PRO	164	33	venom-named:11; serine protease:6; phospholipase:3; apolipophorin:2; cysteine proteinase:2; hyaluronidase:2; venom acid phosphatase:2; allergen:1; carboxylesterase:1; glucosylceramidase:1; hexamerin:1; lysozyme:1; mast cell degranulating peptide:1; melittin:1; nucleobindin:1; omega-conotoxin-like:1; secapin:1; transferrin:1; venom dipeptidyl pep...
VM-V PEP	11	0	
VM-V PRO	197	18	hexamerin:5; venom-named:4; hyaluronidase:2; lysozyme:2; allergen:1; apolipophorin:1; glucosylceramidase:1; nucleobindin:1; serine protease:1; transferrin:1; trehalase:1; venom dipeptidyl peptidase:1

Table S5. GO enrichment summaries and representative terms

S5_GO_summary

group	domain	terms	nominal_p_le_0.05	adjusted_p_le_0.05	max_out_count	out_column	background_column
AM PEP	Cellular component	155	5	0	9	out (9)	All (286)
AM PEP	Molecular function	84	2	0	7	out (7)	All (253)
AM PEP	Biological process	639	36	0	9	out (9)	All (264)
AM PRO	Cellular component	1339	176	62	135	out (135)	All (19970)
AM PRO	Molecular function	2157	279	84	133	out (133)	All (19525)
AM PRO	Biological process	10359	962	164	134	out (134)	All (19828)
VM PEP	Cellular component	156	18	0	2	out (2)	All (192)
VM PEP	Molecular function	89	8	0	2	out (2)	All (184)
VM PEP	Biological process	324	6	0	2	out (2)	All (192)
VM PRO	Cellular component	1521	347	209	167	out (167)	All (7534)
VM PRO	Molecular function	2709	654	208	162	out (162)	All (7337)
VM PRO	Biological process	10950	1856	732	165	out (165)	All (7470)

S5_GO_top_terms

group	domain	go_id	description	out_count	background_count	pvalue	p_adjust	genes
AM PEP	Cellular component	GO:0005764	lysosome	3	24	0.03128 87	0.929686	NP_001011607.1;NP_001011611.2;XP_003249259.2
AM PEP	Cellular component	GO:0031414	N-terminal protein acetyltransferase complex	1	1	0.03146 85	0.929686	XP_001120034.1
AM PEP	Cellular compon	GO:0031417	NatC complex	1	1	0.03146 85	0.929686	XP_001120034.1

	ent							
AM PEP	Cellular component	GO:0044284	mitochondrial crista junction	1	1	0.03146 85	0.929686	XP_001121025.1
AM PEP	Cellular component	GO:0061617	MICOS complex	1	1	0.03146 85	0.929686	XP_001121025.1
AM PEP	Cellular component	GO:0000323	lytic vacuole	3	33	0.07256 46	0.929686	NP_001011607.1;NP_001011611.2;XP_003249259.2
AM PEP	Cellular component	GO:0031248	protein acetyltransferase complex	1	3	0.09177 74	0.929686	XP_001120034.1
AM PEP	Cellular component	GO:0071986	Ragulator complex	1	3	0.09177 74	0.929686	XP_003249259.2
AM PEP	Cellular component	GO:0097505	Rad6-Rad18 complex	1	3	0.09177 74	0.929686	XP_001121462.1
AM PEP	Cellular component	GO:1902493	acetyltransferase complex	1	3	0.09177 74	0.929686	XP_001120034.1

AM PEP	Molecular function	GO:0004566	beta-glucuronidase activity	1	1	0.027668	0.83451	NP_001011607.1
AM PEP	Molecular function	GO:0005104	fibroblast growth factor receptor binding	1	1	0.027668	0.83451	XP_001120034.1
AM PEP	Molecular function	GO:0004860	protein kinase inhibitor activity	1	3	0.081408	0.83451	NP_001011607.1
AM PEP	Molecular function	GO:0009045	xylose isomerase activity	1	3	0.081408	0.83451	XP_001121462.1
AM PEP	Molecular function	GO:0019210	kinase inhibitor activity	1	3	0.081408	0.83451	NP_001011607.1
AM PEP	Molecular function	GO:0001054	RNA polymerase I activity	1	5	0.131882	0.83451	XP_001121462.1
AM PEP	Molecular functi	GO:0015288	porin activity	1	5	0.131882	0.83451	NP_001011607.1

	on							
AMPEP	Molecular function	GO:0022829	wide pore channel activity	1	5	0.131882	0.83451	NP_001011607.1
AMPEP	Molecular function	GO:0003887	DNA-directed DNA polymerase activity	1	6	0.156386	0.83451	XP_001121025.1
AMPEP	Molecular function	GO:0070851	growth factor receptor binding	1	6	0.156386	0.83451	XP_001120034.1
AMPEP	Biological process	GO:0006474	N-terminal protein amino acid acetylation	1	1	0.0340909	0.640709	XP_001120034.1
AMPEP	Biological process	GO:0006606	protein import into nucleus	1	1	0.0340909	0.640709	XP_001120034.1
AMPEP	Biological process	GO:0015716	organic phosphonate transport	1	1	0.0340909	0.640709	XP_001121462.1
AMP	Biological	GO:0017196	N-terminal peptid	1	1	0.0340909	0.640709	XP_001120034.1

EP	process		yl-methionine acetylation					
AMPEP	Biological process	GO:0019389	glucuronoside metabolic process	1	1	0.03409 09	0.640709	NP_001011607.1
AMPEP	Biological process	GO:0019391	glucuronoside catabolic process	1	1	0.03409 09	0.640709	NP_001011607.1
AMPEP	Biological process	GO:0020035	cytoadherence to microvasculature, mediated by symbiont protein	1	1	0.03409 09	0.640709	XP_006563876.1
AMPEP	Biological process	GO:0030212	hyaluronan metabolic process	1	1	0.03409 09	0.640709	NP_001011607.1
AMPEP	Biological process	GO:0030214	hyaluronan catabolic process	1	1	0.03409 09	0.640709	NP_001011607.1
AMPE	Biological pro	GO:0030947	regulation of vascular endot	1	1	0.03409 09	0.640709	XP_001120034.1

P	cess		helial growth factor receptor signaling pathway					
AM PRO	Cellular component	GO:0005576	extracellular region	128	14582	4.9897e-11	6.68121e-08	NP_001011564.1;NP_001011572.1;NP_001011578.1;NP_001011579.1;NP_001011580.1;NP_001011599.1;NP_001011601.1;NP_001011607.1;NP_001011611.2;NP_001011612.1;NP_001011614.1;NP_001011619.1;NP_001011627.1;NP_001019868.1;NP_001035360.1;NP_001090623.1;NP_001104234.1;NP_001119716.1;NP_001153524.1;NP_001165849.1;NP_001171498.1;NP_001229473.1;NP_008082.1;XP_00...
AM PRO	Cellular component	GO:0005788	endoplasmic reticulum lumen	54	3499	5.90132e-10	3.95094e-07	NP_001011572.1;NP_001011614.1;NP_001011627.1;NP_001090623.1;NP_001104234.1;NP_001119716.1;NP_001153524.1;NP_001229473.1;XP_001120293.2;XP_001120594.2;XP_003251367.1;XP_006557450.1;XP_006557712.2;XP_006559569.1;XP_006560620.1;XP_006562600.2;XP_006564557.1;XP_006564744.1;XP_006567277.1;XP_006567533.1;XP_006568873.1;XP_006569897.2;XP_016767538.1;XP...
AM PRO	Cellular component	GO:0043202	lysosomal lumen	27	1468	1.55108e-06	0.0006923	NP_001011607.1;NP_001011619.1;XP_001120140.3;XP_001120594.2;XP_001121565.2;XP_006562600.2;XP_006562719.1;XP_016767538.1;XP_016770902.1;XP_016771167.1;XP_016772427.1;XP_026295178.1;XP_026296694.1;XP_026297212.1;XP_026298883.1;XP_026299604.1;XP_026299738.1;XP_026300654.1;XP_026300858.1;XP_392381.3;XP_392617.4;XP_392857.2;XP_395852.4;XP_623282.2;XP...
AM PRO	Cellular component	GO:0097524	sperm plasma membrane	10	230	3.78802e-06	0.0011890 2	XP_001120594.2;XP_006560620.1;XP_016772088.2;XP_016772200.1;XP_026296056.1;XP_026296405.1;XP_026300310.1;XP_026300525.1;XP_395614.3;XP_395852.4
AM PRO	Cellular component	GO:0097486	multivesicular body lumen	4	17	4.43996e-06	0.0011890 2	XP_006562600.2;XP_006562719.1;XP_392381.3;XP_392857.2
AM PR	Cellular comp	GO:0031906	late endosome lumen	4	18	5.67862e-06	0.0012672 8	XP_006562600.2;XP_006562719.1;XP_392381.3;XP_392857.2

O	onent							
AMPRO	Cellular component	GO:0033181	plasma membrane proton-transporting V-type ATPase complex	7	108	8.72244e-06	0.0016684 8	XP_001120594.2;XP_001121483.3;XP_006560620.1;XP_016772088.2;XP_016772200.1;XP_026296056.1;XP_026300310.1
AMPRO	Cellular component	GO:0031983	vesicle lumen	50	4191	1.3001e-05	0.0021760 4	NP_001011572.1;NP_001011578.1;NP_001011607.1;NP_001011614.1;NP_001090623.1;NP_001153524.1;NP_001229473.1;XP_001120140.3;XP_001120293.2;XP_001120594.2;XP_001121565.2;XP_003249289.2;XP_006560620.1;XP_006562600.2;XP_006564557.1;XP_006567452.1;XP_006568105.2;XP_006568978.1;XP_006569897.2;XP_016769016.1;XP_016771021.1;XP_016771167.1;XP_016772088.2;XP...
AMPRO	Cellular component	GO:0001405	presequence translocase-associated import motor	7	125	2.26813e-05	0.0030594 6	NP_001153524.1;XP_001120594.2;XP_006560620.1;XP_016772088.2;XP_016772200.1;XP_026296056.1;XP_026300310.1
AMPRO	Cellular component	GO:0016471	vacuolar proton-transporting V-type ATPase complex	9	226	2.36191e-05	0.0030594 6	NP_001090623.1;XP_001120594.2;XP_001121483.3;XP_006558237.1;XP_006560620.1;XP_016772088.2;XP_016772200.1;XP_026296056.1;XP_026300310.1
AMPRO	Molecular functi	GO:0015207	adenine transmembrane transp	11	190	7.45803e-08	0.0001608 7	NP_001011564.1;NP_001011579.1;NP_001011580.1;NP_001011599.1;NP_001011601.1;NP_001019868.1;XP_006560620.1;XP_016772088.2;XP_016772200.1;XP_026296056.1;XP_026300310.1

	on		orter activity					
AM PRO	Molecular function	GO:0004029	aldehyde dehydrogenase (NAD) activity	13	340	5.88444e-07	0.000634637	NP_001011614.1;XP_001120293.2;XP_001120594.2;XP_006560620.1;XP_006563998.1;XP_006565513.1;XP_016767722.1;XP_016772088.2;XP_016772200.1;XP_026296056.1;XP_026300310.1;XP_394209.2;XP_394210.5
AM PRO	Molecular function	GO:0005345	purine nucleobase transmembrane transporter activity	11	265	2.04868e-06	0.001473	NP_001011564.1;NP_001011579.1;NP_001011580.1;NP_001011599.1;NP_001011601.1;NP_001019868.1;XP_006560620.1;XP_016772088.2;XP_016772200.1;XP_026296056.1;XP_026300310.1
AM PRO	Molecular function	GO:0046593	mandelonitrile lyase activity	5	46	1.48976e-05	0.0080335 2	XP_006565513.1;XP_016767722.1;XP_026300525.1;XP_394209.2;XP_394210.5
AM PRO	Molecular function	GO:0015205	nucleobase transmembrane transporter activity	11	357	3.38225e-05	0.0110568	NP_001011564.1;NP_001011579.1;NP_001011580.1;NP_001011599.1;NP_001011601.1;NP_001019868.1;XP_006560620.1;XP_016772088.2;XP_016772200.1;XP_026296056.1;XP_026300310.1
AM PRO	Molecular function	GO:0047735	cellobiose dehydrogenase (acceptor) activity	4	28	3.71108e-05	0.0110568	XP_006565513.1;XP_016767722.1;XP_394209.2;XP_394210.5
AM	Molecul	GO:00170	sulfonylurea recept	7	134	3.71727	0.0110568	XP_001120594.2;XP_006560620.1;XP_016772088.2;XP_016772200.1;XP_026296056.1;XP_026300310.1;XP_395614.3

PRO	ar function	98	or binding			e-05		
AM PRO	Molecular function	GO:0097005	adipokinetic hormone receptor binding	2	2	4.60538e-05	0.0110568	NP_001011619.1;XP_026300654.1
AM PRO	Molecular function	GO:0000721	(R,R)-butanediol dehydrogenase activity	6	100	6.29547e-05	0.0110568	XP_001120594.2;XP_006560620.1;XP_016772088.2;XP_016772200.1;XP_026296056.1;XP_026300310.1
AM PRO	Molecular function	GO:0032564	dATP binding	7	148	6.99462e-05	0.0110568	XP_001120594.2;XP_006560620.1;XP_016772088.2;XP_016772200.1;XP_026296056.1;XP_026300310.1;XP_395614.3
AM PRO	Biological process	GO:0048647	polyphenic determination	6	29	3.55881e-08	9.21643e-05	NP_001011564.1;NP_001011579.1;NP_001011580.1;NP_001011599.1;NP_001011601.1;NP_001019868.1
AM PRO	Biological process	GO:0048648	caste determination	6	29	3.55881e-08	9.21643e-05	NP_001011564.1;NP_001011579.1;NP_001011580.1;NP_001011599.1;NP_001011601.1;NP_001019868.1
AM PRO	Biological process	GO:0048650	caste determination, influence by environment	6	29	3.55881e-08	9.21643e-05	NP_001011564.1;NP_001011579.1;NP_001011580.1;NP_001011599.1;NP_001011601.1;NP_001019868.1

			al factors					
AM PRO	Biological process	GO:0048651	polyphenic determination, influence by environmental factors	6	29	3.55881e-08	9.21643e-05	NP_001011564.1;NP_001011579.1;NP_001011580.1;NP_001011599.1;NP_001011601.1;NP_001019868.1
AM PRO	Biological process	GO:0035007	regulation of melanization defense response	9	123	1.54038e-07	0.000319135	NP_001011627.1;NP_001104234.1;XP_001120594.2;XP_006560620.1;XP_016772088.2;XP_026296056.1;XP_026299604.1;XP_026300310.1;XP_026300858.1
AM PRO	Biological process	GO:0010755	regulation of plasminogen activation	13	371	1.43603e-06	0.0023913 2	NP_001011572.1;XP_001120594.2;XP_006560620.1;XP_006562600.2;XP_016772088.2;XP_016772200.1;XP_026296056.1;XP_026296936.1;XP_026298462.1;XP_026299604.1;XP_026300310.1;XP_026300858.1;XP_392381.3
AM PRO	Biological process	GO:0035008	positive regulation of melanization defense response	7	84	1.61592e-06	0.00239132	NP_001011627.1;NP_001104234.1;XP_001120594.2;XP_006560620.1;XP_016772088.2;XP_026296056.1;XP_026300310.1
AM PRO	Biological process	GO:0007311	maternal specification of dorsal/ventral	7	89	2.39032e-06	0.00309517	XP_001120594.2;XP_006560620.1;XP_006562000.1;XP_016772088.2;XP_016773517.2;XP_026296056.1;XP_026300310.1

			axis, oocyte, germ-line encoded					
AMPRO	Biological process	GO:0016486	peptide hormone processing	18	746	2.85949e-06	0.00329127	NP_001090623.1;NP_001119716.1;XP_001120594.2;XP_006558237.1;XP_006560620.1;XP_006562600.2;XP_006567452.1;XP_006569897.2;XP_016772088.2;XP_016772200.1;XP_026296056.1;XP_026296936.1;XP_026298462.1;XP_026299604.1;XP_026300310.1;XP_026300858.1;XP_392381.3;XP_392857.2
AMPRO	Biological process	GO:0045040	protein import into mitochondrial outer membrane	8	146	6.79724e-06	0.00673147	XP_001120594.2;XP_006560620.1;XP_006564557.1;XP_016772088.2;XP_016772200.1;XP_026296056.1;XP_026300310.1;XP_395614.3
VMPEP	Cellular component	GO:0000243	commitment complex	1	1	0.0104167	0.303092	Gene.585::TRINITY_DN12396_c0_g2::g.585::m.585
VMPEP	Cellular component	GO:0005685	U1 snRNP	1	2	0.0207788	0.303092	Gene.585::TRINITY_DN12396_c0_g2::g.585::m.585
VMPEP	Cellular component	GO:0005687	U4 snRNP	1	2	0.0207788	0.303092	Gene.585::TRINITY_DN12396_c0_g2::g.585::m.585
VMPE	Cellular comp	GO:0005689	U12-type spliceosomal	1	2	0.0207788	0.303092	Gene.585::TRINITY_DN12396_c0_g2::g.585::m.585

P	onent		complex					
VM PEP	Cellular component	GO:0008328	ionotropic glutamate receptor complex	1	2	0.0207788	0.303092	Gene.585::TRINITY_DN12396_c0_g2::g.585::m.585
VM PEP	Cellular component	GO:0032281	AMPA glutamate receptor complex	1	2	0.0207788	0.303092	Gene.585::TRINITY_DN12396_c0_g2::g.585::m.585
VM PEP	Cellular component	GO:0034719	SMN-Sm protein complex	1	2	0.0207788	0.303092	Gene.585::TRINITY_DN12396_c0_g2::g.585::m.585
VM PEP	Cellular component	GO:0005682	U5 snRNP	1	3	0.0310864	0.303092	Gene.585::TRINITY_DN12396_c0_g2::g.585::m.585
VM PEP	Cellular component	GO:0005686	U2 snRNP	1	3	0.0310864	0.303092	Gene.585::TRINITY_DN12396_c0_g2::g.585::m.585
VM PEP	Cellular component	GO:0034715	pICln-Sm protein complex	1	3	0.0310864	0.303092	Gene.585::TRINITY_DN12396_c0_g2::g.585::m.585
VM P	Molecular	GO:0004070	aspartate carbamoylt	1	2	0.0216797	0.321583	Gene.585::TRINITY_DN12396_c0_g2::g.585::m.585

EP	function		ransferase activity					
VM PEP	Molecular function	GO:0004970	ionotropic glutamate receptor activity	1	2	0.02167 97	0.321583	Gene.585::TRINITY_DN12396_c0_g2::g.585::m.585
VM PEP	Molecular function	GO:0004971	AMPA glutamate receptor activity	1	2	0.02167 97	0.321583	Gene.585::TRINITY_DN12396_c0_g2::g.585::m.585
VM PEP	Molecular function	GO:0016615	malate dehydrogenase activity	1	2	0.02167 97	0.321583	Gene.59::TRINITY_DN10461_c0_g1::g.59::m.59
VM PEP	Molecular function	GO:0030060	L-malate dehydrogenase activity	1	2	0.02167 97	0.321583	Gene.59::TRINITY_DN10461_c0_g1::g.59::m.59
VM PEP	Molecular function	GO:0046554	malate dehydrogenase (NADP+) activity	1	2	0.02167 97	0.321583	Gene.59::TRINITY_DN10461_c0_g1::g.59::m.59
VM PEP	Molecular functi	GO:0008066	glutamate receptor activity	1	3	0.03243 05	0.354842	Gene.585::TRINITY_DN12396_c0_g2::g.585::m.585

	on							
VMPEP	Molecular function	GO:0016743	carboxyl- or carbamoyltransferase activity	1	4	0.04312 19	0.354842	Gene.585::TRINITY_DN12396_c0_g2::g.585::m.585
VMPEP	Molecular function	GO:0004308	exo-alpha-sialidase activity	1	6	0.06432 64	0.354842	Gene.59::TRINITY_DN10461_c0_g1::g.59::m.59
VMPEP	Molecular function	GO:0052794	exo-alpha-(2->3)-sialidase activity	1	6	0.06432 64	0.354842	Gene.59::TRINITY_DN10461_c0_g1::g.59::m.59
VMPEP	Biological process	GO:0002182	cytoplasmic translational elongation	1	2	0.02077 88	0.730698	Gene.585::TRINITY_DN12396_c0_g2::g.585::m.585
VMPEP	Biological process	GO:0006108	malate metabolic process	1	2	0.02077 88	0.730698	Gene.59::TRINITY_DN10461_c0_g1::g.59::m.59
VMPEP	Biological process	GO:0060992	response to fungicide	1	2	0.02077 88	0.730698	Gene.585::TRINITY_DN12396_c0_g2::g.585::m.585
VMP	Biological	GO:1904400	response to Thyroid	1	3	0.03108 64	0.730698	Gene.585::TRINITY_DN12396_c0_g2::g.585::m.585

EP	process		stimulating hormone					
VM PEP	Biological process	GO:1904401	cellular response to Thyroid stimulating hormone	1	3	0.03108864	0.730698	Gene.585::TRINITY_DN12396_c0_g2::g.585::m.585
VM PEP	Biological process	GO:0006107	oxaloacetate metabolic process	1	4	0.04133394	0.730698	Gene.59::TRINITY_DN10461_c0_g1::g.59::m.59
VM PEP	Biological process	GO:0000387	spliceosomal snRNP assembly	1	5	0.0515538	0.730698	Gene.585::TRINITY_DN12396_c0_g2::g.585::m.585
VM PEP	Biological process	GO:0006222	UMP biosynthetic process	1	5	0.0515538	0.730698	Gene.585::TRINITY_DN12396_c0_g2::g.585::m.585
VM PEP	Biological process	GO:0044205	'de novo' UMP biosynthetic process	1	5	0.0515538	0.730698	Gene.585::TRINITY_DN12396_c0_g2::g.585::m.585
VM PEP	Biological process	GO:0046049	UMP metabolic process	1	5	0.0515538	0.730698	Gene.585::TRINITY_DN12396_c0_g2::g.585::m.585

VM PRO	Cellular component	GO:0005576	extracellular region	156	5058	1.59134e-16	2.42043e-13	Gene.1004::TRINITY_DN12660_c0_g2::g.1004::m.1004;Gene.10237::TRINITY_DN14623_c0_g10::g.10237::m.10237;Gene.10266::TRINITY_DN14626_c0_g5::g.10266::m.10266;Gene.10655::TRINITY_DN14700_c1_g7::g.10655::m.10655;Gene.10698::TRINITY_DN14709_c4_g5::g.10698::m.10698;Gene.1116::TRINITY_DN12684_c1_g3::g.1116::m.1116;Gene.11221::TRINITY_DN14818_c2_g3::g.112...
VM PRO	Cellular component	GO:0005615	extracellular space	131	3689	2.4082e-15	1.83143e-12	Gene.1004::TRINITY_DN12660_c0_g2::g.1004::m.1004;Gene.10237::TRINITY_DN14623_c0_g10::g.10237::m.10237;Gene.10266::TRINITY_DN14626_c0_g5::g.10266::m.10266;Gene.10655::TRINITY_DN14700_c1_g7::g.10655::m.10655;Gene.10698::TRINITY_DN14709_c4_g5::g.10698::m.10698;Gene.11221::TRINITY_DN14818_c2_g3::g.11221::m.11221;Gene.11325::TRINITY_DN14842_c2_g3::g....
VM PRO	Cellular component	GO:0070062	extracellular exosome	125	3419	4.8314e-15	2.26643e-12	Gene.10237::TRINITY_DN14623_c0_g10::g.10237::m.10237;Gene.10266::TRINITY_DN14626_c0_g5::g.10266::m.10266;Gene.10655::TRINITY_DN14700_c1_g7::g.10655::m.10655;Gene.10698::TRINITY_DN14709_c4_g5::g.10698::m.10698;Gene.11221::TRINITY_DN14818_c2_g3::g.11221::m.11221;Gene.11325::TRINITY_DN14842_c2_g3::g.11325::m.11325;Gene.11514::TRINITY_DN14882_c1_g3:...
VM PRO	Cellular component	GO:1903561	extracellular vesicle	125	3429	6.33602e-15	2.26643e-12	Gene.10237::TRINITY_DN14623_c0_g10::g.10237::m.10237;Gene.10266::TRINITY_DN14626_c0_g5::g.10266::m.10266;Gene.10655::TRINITY_DN14700_c1_g7::g.10655::m.10655;Gene.10698::TRINITY_DN14709_c4_g5::g.10698::m.10698;Gene.11221::TRINITY_DN14818_c2_g3::g.11221::m.11221;Gene.11325::TRINITY_DN14842_c2_g3::g.11325::m.11325;Gene.11514::TRINITY_DN14882_c1_g3:...
VM PRO	Cellular component	GO:0065010	extracellular membrane-bounded organelle	125	3435	7.45045e-15	2.26643e-12	Gene.10237::TRINITY_DN14623_c0_g10::g.10237::m.10237;Gene.10266::TRINITY_DN14626_c0_g5::g.10266::m.10266;Gene.10655::TRINITY_DN14700_c1_g7::g.10655::m.10655;Gene.10698::TRINITY_DN14709_c4_g5::g.10698::m.10698;Gene.11221::TRINITY_DN14818_c2_g3::g.11221::m.11221;Gene.11325::TRINITY_DN14842_c2_g3::g.11325::m.11325;Gene.11514::TRINITY_DN14882_c1_g3:...
VM PRO	Cellular component	GO:0043230	extracellular organelle	125	3445	9.74977e-15	2.47157e-12	Gene.10237::TRINITY_DN14623_c0_g10::g.10237::m.10237;Gene.10266::TRINITY_DN14626_c0_g5::g.10266::m.10266;Gene.10655::TRINITY_DN14700_c1_g7::g.10655::m.10655;Gene.10698::TRINITY_DN14709_c4_g5::g.10698::m.10698;Gene.11221::TRINITY_DN14818_c2_g3::g.11221::m.11221;Gene.11325::TRINITY_DN14842_c2_g3::g.11325::m.11325;Gene.11514::TRINITY_DN14882_c1_g3:...
VM PRO	Cellular component	GO:0005618	cell wall	76	1546	1.8292e-13	3.9746e-11	Gene.10237::TRINITY_DN14623_c0_g10::g.10237::m.10237;Gene.10266::TRINITY_DN14626_c0_g5::g.10266::m.10266;Gene.10698::TRINITY_DN14709_c4_g5::g.10698::m.10698;Gene.11221::TRINITY_DN14818_c2_g3::g.11221::m.11221;Gene.11514::TRINITY_DN14882_c1_g3::g.11514::m.11514;Gene.11564::TRINITY_DN14890_c2_g15::g.11564::m.11564;Gene.1202::TRINITY_DN12702_c0_g13...

VM PRO	Cellular component	GO:0005788	endoplasmic reticulum lumen	68	1286	2.41284e-13	4.58742e-11	Gene.10237::TRINITY_DN14623_c0_g10::g.10237::m.10237;Gene.10266::TRINITY_DN14626_c0_g5::g.10266::m.10266;Gene.10698::TRINITY_DN14709_c4_g5::g.10698::m.10698;Gene.11514::TRINITY_DN14882_c1_g3::g.11514::m.11514;Gene.1202::TRINITY_DN12702_c0_g13::g.1202::m.1202;Gene.1206::TRINITY_DN12702_c0_g2::g.1206::m.1206;Gene.12239::TRINITY_DN15036_c0_g10::g.1...
VM PRO	Cellular component	GO:0044421	extracellular region part	141	4453	8.12011e-13	1.3723e-10	Gene.1004::TRINITY_DN12660_c0_g2::g.1004::m.1004;Gene.10237::TRINITY_DN14623_c0_g10::g.10237::m.10237;Gene.10266::TRINITY_DN14626_c0_g5::g.10266::m.10266;Gene.10655::TRINITY_DN14700_c1_g7::g.10655::m.10655;Gene.10698::TRINITY_DN14709_c4_g5::g.10698::m.10698;Gene.11221::TRINITY_DN14818_c2_g3::g.11221::m.11221;Gene.11325::TRINITY_DN14842_c2_g3::g....
VM PRO	Cellular component	GO:0030312	external encapsulating structure	86	1983	2.16363e-12	3.29087e-10	Gene.10237::TRINITY_DN14623_c0_g10::g.10237::m.10237;Gene.10266::TRINITY_DN14626_c0_g5::g.10266::m.10266;Gene.10698::TRINITY_DN14709_c4_g5::g.10698::m.10698;Gene.1116::TRINITY_DN12684_c1_g3::g.1116::m.1116;Gene.11221::TRINITY_DN14818_c2_g3::g.11221::m.11221;Gene.11514::TRINITY_DN14882_c1_g3::g.11514::m.11514;Gene.11564::TRINITY_DN14890_c2_g15::g...
VM PRO	Molecular function	GO:0008194	UDP-glycosyltransferase activity	32	438	1.18096e-09	3.19922e-06	Gene.10266::TRINITY_DN14626_c0_g5::g.10266::m.10266;Gene.11325::TRINITY_DN14842_c2_g3::g.11325::m.11325;Gene.11514::TRINITY_DN14882_c1_g3::g.11514::m.11514;Gene.12728::TRINITY_DN15134_c4_g5::g.12728::m.12728;Gene.13010::TRINITY_DN15192_c1_g13::g.13010::m.13010;Gene.14440::TRINITY_DN15472_c0_g1::g.14440::m.14440;Gene.15333::TRINITY_DN15642_c1_g1:...
VM PRO	Molecular function	GO:0016757	transferase activity, transferring glycosyl groups	62	1457	3.34162e-08	3.17294e-05	Gene.10266::TRINITY_DN14626_c0_g5::g.10266::m.10266;Gene.11221::TRINITY_DN14818_c2_g3::g.11221::m.11221;Gene.11325::TRINITY_DN14842_c2_g3::g.11325::m.11325;Gene.11514::TRINITY_DN14882_c1_g3::g.11514::m.11514;Gene.1202::TRINITY_DN12702_c0_g13::g.1202::m.1202;Gene.1206::TRINITY_DN12702_c0_g2::g.1206::m.1206;Gene.12728::TRINITY_DN15134_c4_g5::g.127...
VM PRO	Molecular function	GO:0005507	copper ion binding	50	1046	3.51378e-08	3.17294e-05	Gene.11221::TRINITY_DN14818_c2_g3::g.11221::m.11221;Gene.11564::TRINITY_DN14890_c2_g15::g.11564::m.11564;Gene.1202::TRINITY_DN12702_c0_g13::g.1202::m.1202;Gene.1206::TRINITY_DN12702_c0_g2::g.1206::m.1206;Gene.12391::TRINITY_DN15068_c2_g14::g.12391::m.12391;Gene.12728::TRINITY_DN15134_c4_g5::g.12728::m.12728;Gene.12799::TRINITY_DN15149_c1_g7::g.1...
VM PR	Molecular fun	GO:0016651	oxidoreductase activity,	52	1137	7.38366e-08	5.00058e-05	Gene.10698::TRINITY_DN14709_c4_g5::g.10698::m.10698;Gene.11221::TRINITY_DN14818_c2_g3::g.11221::m.11221;Gene.11514::TRINITY_DN14882_c1_g3::g.11514::m.11514;Gene.11564::TRINITY_DN14890_c2_g15::g.11564::m.11564;Gene.12568::TRINITY_DN15103_c1_g5::g.12568::m.12568;Gene.14347::TRINITY_DN15450_c1_g4::g.14347::m.14347;Gene.14365::TRINITY_DN15454_c1_g6:...

O	ction		acting on NAD(P)H					
VM PRO	Molecular function	GO:0016491	oxidoreductase activity	115	3750	1.54737e-07	8.38363e-05	Gene.10237::TRINITY_DN14623_c0_g10::g.10237::m.10237;Gene.10266::TRINITY_DN14626_c0_g5::g.10266::m.10266;Gene.10698::TRINITY_DN14709_c4_g5::g.10698::m.10698;Gene.1116::TRINITY_DN12684_c1_g3::g.1116::m.1116;Gene.11221::TRINITY_DN14818_c2_g3::g.11221::m.11221;Gene.11514::TRINITY_DN14882_c1_g3::g.11514::m.11514;Gene.11564::TRINITY_DN14890_c2_g15::g...
VM PRO	Molecular function	GO:0008084	imaginal disc growth factor receptor binding	5	8	2.61971e-07	0.00011828	Gene.1202::TRINITY_DN12702_c0_g13::g.1202::m.1202;Gene.1206::TRINITY_DN12702_c0_g2::g.1206::m.1206;Gene.12799::TRINITY_DN15149_c1_g7::g.12799::m.12799;Gene.13945::TRINITY_DN15367_c2_g6::g.13945::m.13945;Gene.4068::TRINITY_DN13303_c2_g4::g.4068::m.4068
VM PRO	Molecular function	GO:0016787	hydrolase activity	136	4898	5.15275e-07	0.000199412	Gene.1004::TRINITY_DN12660_c0_g2::g.1004::m.1004;Gene.10237::TRINITY_DN14623_c0_g10::g.10237::m.10237;Gene.10266::TRINITY_DN14626_c0_g5::g.10266::m.10266;Gene.10655::TRINITY_DN14700_c1_g7::g.10655::m.10655;Gene.10698::TRINITY_DN14709_c4_g5::g.10698::m.10698;Gene.1116::TRINITY_DN12684_c1_g3::g.1116::m.1116;Gene.11221::TRINITY_DN14818_c2_g3::g.112...
VM PRO	Molecular function	GO:0051087	chaperone binding	38	751	6.37565e-07	0.000215896	Gene.10698::TRINITY_DN14709_c4_g5::g.10698::m.10698;Gene.11221::TRINITY_DN14818_c2_g3::g.11221::m.11221;Gene.12568::TRINITY_DN15103_c1_g5::g.12568::m.12568;Gene.14944::TRINITY_DN15571_c2_g1::g.14944::m.14944;Gene.15383::TRINITY_DN15652_c2_g4::g.15383::m.15383;Gene.15771::TRINITY_DN15718_c1_g9::g.15771::m.15771;Gene.15917::TRINITY_DN15743_c2_g11:...
VM PRO	Molecular function	GO:0016758	transferase activity, transferring hexosyl groups	47	1048	7.36213e-07	0.0002216	Gene.10266::TRINITY_DN14626_c0_g5::g.10266::m.10266;Gene.11221::TRINITY_DN14818_c2_g3::g.11221::m.11221;Gene.11325::TRINITY_DN14842_c2_g3::g.11325::m.11325;Gene.11514::TRINITY_DN14882_c1_g3::g.11514::m.11514;Gene.12728::TRINITY_DN15134_c4_g5::g.12728::m.12728;Gene.13010::TRINITY_DN15192_c1_g13::g.13010::m.13010;Gene.14440::TRINITY_DN15472_c0_g1:...
VM PR	Molecular fun	GO:0035251	UDP-glucosyltransferase	13	112	9.38155e-07	0.000236982	Gene.12728::TRINITY_DN15134_c4_g5::g.12728::m.12728;Gene.13010::TRINITY_DN15192_c1_g13::g.13010::m.13010;Gene.14440::TRINITY_DN15472_c0_g1::g.14440::m.14440;Gene.15333::TRINITY_DN15642_c1_g1::g.15333::m.15333;Gene.15771::TRINITY_DN15718_c1_g9::g.15771::m.15771;Gene.2374::TRINITY_DN12965_c2_g1::g.2374::m.2374;Gene.3563::TRINITY_DN13203_c2_g2::g.3...

O	ction		activity					
VMPRO	Biological process	GO:1901565	organonitrogen compound catabolic process	83	2107	1.12242e-09	7.17265e-06	Gene.1004::TRINITY_DN12660_c0_g2::g.1004::m.1004;Gene.10237::TRINITY_DN14623_c0_g10::g.10237::m.10237;Gene.10266::TRINITY_DN14626_c0_g5::g.10266::m.10266;Gene.10655::TRINITY_DN14700_c1_g7::g.10655::m.10655;Gene.10698::TRINITY_DN14709_c4_g5::g.10698::m.10698;Gene.11221::TRINITY_DN14818_c2_g3::g.11221::m.11221;Gene.11325::TRINITY_DN14842_c2_g3::g....
VMPRO	Biological process	GO:0009617	response to bacterium	103	2943	1.31007e-09	7.17265e-06	Gene.1004::TRINITY_DN12660_c0_g2::g.1004::m.1004;Gene.10237::TRINITY_DN14623_c0_g10::g.10237::m.10237;Gene.10698::TRINITY_DN14709_c4_g5::g.10698::m.10698;Gene.11221::TRINITY_DN14818_c2_g3::g.11221::m.11221;Gene.11514::TRINITY_DN14882_c1_g3::g.11514::m.11514;Gene.11564::TRINITY_DN14890_c2_g15::g.11564::m.11564;Gene.1202::TRINITY_DN12702_c0_g13::g...
VMPRO	Biological process	GO:0002252	immune effector process	114	3481	3.03295e-09	8.5147e-06	Gene.10237::TRINITY_DN14623_c0_g10::g.10237::m.10237;Gene.10266::TRINITY_DN14626_c0_g5::g.10266::m.10266;Gene.10698::TRINITY_DN14709_c4_g5::g.10698::m.10698;Gene.11221::TRINITY_DN14818_c2_g3::g.11221::m.11221;Gene.11311::TRINITY_DN14838_c0_g4::g.11311::m.11311;Gene.11325::TRINITY_DN14842_c2_g3::g.11325::m.11325;Gene.11514::TRINITY_DN14882_c1_g3:...
VMPRO	Biological process	GO:0006979	response to oxidative stress	93	2554	3.11039e-09	8.5147e-06	Gene.10237::TRINITY_DN14623_c0_g10::g.10237::m.10237;Gene.10698::TRINITY_DN14709_c4_g5::g.10698::m.10698;Gene.1116::TRINITY_DN12684_c1_g3::g.1116::m.1116;Gene.11221::TRINITY_DN14818_c2_g3::g.11221::m.11221;Gene.11311::TRINITY_DN14838_c0_g4::g.11311::m.11311;Gene.11325::TRINITY_DN14842_c2_g3::g.11325::m.11325;Gene.11514::TRINITY_DN14882_c1_g3::g....
VMPRO	Biological process	GO:0006955	immune response	125	4119	2.37608e-08	4.24269e-05	Gene.10237::TRINITY_DN14623_c0_g10::g.10237::m.10237;Gene.10266::TRINITY_DN14626_c0_g5::g.10266::m.10266;Gene.10698::TRINITY_DN14709_c4_g5::g.10698::m.10698;Gene.11221::TRINITY_DN14818_c2_g3::g.11221::m.11221;Gene.11311::TRINITY_DN14838_c0_g4::g.11311::m.11311;Gene.11514::TRINITY_DN14882_c1_g3::g.11514::m.11514;Gene.11564::TRINITY_DN14890_c2_g15...
VMPRO	Biological process	GO:0042742	defense response to bacterium	82	2199	2.58399e-08	4.24269e-05	Gene.1004::TRINITY_DN12660_c0_g2::g.1004::m.1004;Gene.10237::TRINITY_DN14623_c0_g10::g.10237::m.10237;Gene.10698::TRINITY_DN14709_c4_g5::g.10698::m.10698;Gene.11221::TRINITY_DN14818_c2_g3::g.11221::m.11221;Gene.11514::TRINITY_DN14882_c1_g3::g.11514::m.11514;Gene.11564::TRINITY_DN14890_c2_g15::g.11564::m.11564;Gene.12391::TRINITY_DN15068_c2_g14::...
VMP	Biological	GO:1901135	carbohydrate deriva	118	3781	2.71222e-	4.24269e-05	Gene.1004::TRINITY_DN12660_c0_g2::g.1004::m.1004;Gene.10237::TRINITY_DN14623_c0_g10::g.10237::m.10237;Gene.10266::TRINITY_DN14626_c0_g5::g.10266::m.10266;Gene.10655::TRINITY_DN14700_c1_g7::g.10655::m.10655;Gene.10698::TRINITY_DN14709_c4_g5::g.10698::m.10698;Gene.11221::TRINITY_DN14818_c2_g3::g.11221::m.11221;Gene.11325::TRINITY_DN14842_c2_g3::g....

RO	process		tive metabolic process			08		
VM PRO	Biological process	GO:0051707	response to other organism	115	3668	4.86377e-08	6.14625e-05	Gene.1004::TRINITY_DN12660_c0_g2::g.1004::m.1004;Gene.10237::TRINITY_DN14623_c0_g10::g.10237::m.10237;Gene.10266::TRINITY_DN14626_c0_g5::g.10266::m.10266;Gene.10698::TRINITY_DN14709_c4_g5::g.10698::m.10698;Gene.11221::TRINITY_DN14818_c2_g3::g.11221::m.11221;Gene.11325::TRINITY_DN14842_c2_g3::g.11325::m.11325;Gene.11514::TRINITY_DN14882_c1_g3::g....
VM PRO	Biological process	GO:0043207	response to external biotic stimulus	115	3670	5.05171e-08	6.14625e-05	Gene.1004::TRINITY_DN12660_c0_g2::g.1004::m.1004;Gene.10237::TRINITY_DN14623_c0_g10::g.10237::m.10237;Gene.10266::TRINITY_DN14626_c0_g5::g.10266::m.10266;Gene.10698::TRINITY_DN14709_c4_g5::g.10698::m.10698;Gene.11221::TRINITY_DN14818_c2_g3::g.11221::m.11221;Gene.11325::TRINITY_DN14842_c2_g3::g.11325::m.11325;Gene.11514::TRINITY_DN14882_c1_g3::g....
VM PRO	Biological process	GO:0010035	response to inorganic substance	102	3093	7.40442e-08	7.16214e-05	Gene.10237::TRINITY_DN14623_c0_g10::g.10237::m.10237;Gene.10698::TRINITY_DN14709_c4_g5::g.10698::m.10698;Gene.1116::TRINITY_DN12684_c1_g3::g.1116::m.1116;Gene.11221::TRINITY_DN14818_c2_g3::g.11221::m.11221;Gene.11311::TRINITY_DN14838_c0_g4::g.11311::m.11311;Gene.11514::TRINITY_DN14882_c1_g3::g.11514::m.11514;Gene.11564::TRINITY_DN14890_c2_g15::g...

S5_GO_level2_top_terms

group	level1_term	level2_go_id	level2_term	count
AM PEP	Biological Process	GO:0050896	response to stimulus	7
AM PEP	Biological Process	GO:0009987	cellular process	7
AM PEP	Cellular Component	GO:0043226	organelle	7
AM PEP	Cellular Component	GO:0044464	cell part	7
AM PEP	Cellular Component	GO:0005623	cell	7
AM PEP	Biological Process	GO:0044699	single-organism process	7
AM PEP	Biological Process	GO:0051704	multi-organism process	6
AM PEP	Cellular Component	GO:0044422	organelle part	6
AM PEP	Cellular Component	GO:0032991	macromolecular complex	6
AM PEP	Cellular Component	GO:0044425	membrane part	6
AM PEP	Cellular Component	GO:0016020	membrane	6
AM PEP	Biological Process	GO:0008152	metabolic process	6
AM PRO	Biological Process	GO:0044699	single-organism process	130
AM PRO	Biological Process	GO:0009987	cellular process	130
AM PRO	Cellular Component	GO:0044464	cell part	129
AM PRO	Cellular Component	GO:0005623	cell	129
AM PRO	Cellular Component	GO:0005576	extracellular region	128
AM PRO	Biological Process	GO:0008152	metabolic process	128

AM PRO	Molecular Function	GO:0005488	binding	128
AM PRO	Cellular Component	GO:0043226	organelle	126
AM PRO	Biological Process	GO:0065007	biological regulation	126
AM PRO	Biological Process	GO:0050896	response to stimulus	126
AM PRO	Biological Process	GO:0050789	regulation of biological process	123
AM PRO	Cellular Component	GO:0016020	membrane	122
VM PEP	Biological Process	GO:0051704	multi-organism process	2
VM PEP	Biological Process	GO:0008152	metabolic process	2
VM PEP	Cellular Component	GO:0005576	extracellular region	2
VM PEP	Biological Process	GO:0009987	cellular process	2
VM PEP	Biological Process	GO:0044699	single-organism process	2
VM PEP	Cellular Component	GO:0044464	cell part	2
VM PEP	Cellular Component	GO:0043226	organelle	2
VM PEP	Cellular Component	GO:0016020	membrane	2
VM PEP	Molecular Function	GO:0003824	catalytic activity	2
VM PEP	Molecular Function	GO:0005488	binding	2
VM PEP	Cellular Component	GO:0044422	organelle part	2
VM PEP	Cellular Component	GO:0005623	cell	2
VM PRO	Biological Process	GO:0044699	single-organism process	162
VM PRO	Biological Process	GO:0009987	cellular process	160
VM PRO	Cellular Component	GO:0005623	cell	160
VM PRO	Cellular Component	GO:0044464	cell part	160
VM PRO	Molecular Function	GO:0005488	binding	159
VM PRO	Cellular Component	GO:0005576	extracellular region	156
VM PRO	Biological Process	GO:0065007	biological regulation	154
VM PRO	Cellular Component	GO:0043226	organelle	154
VM PRO	Biological Process	GO:0008152	metabolic process	153
VM PRO	Cellular Component	GO:0016020	membrane	153
VM PRO	Biological Process	GO:0032502	developmental process	153
VM PRO	Biological Process	GO:0050789	regulation of biological process	152

Table S6. KEGG enrichment summaries and representative pathways

S6_KEGG_summary

group	pathways	nominal_p_le_0.05	q_le_0.05	max_out_count	out_column	background_column	class_summary
AM PEP	8	1	0	1	out (2)	All (135)	Metabolism:3; Human Diseases:2; Genetic Information Processing:1; Environmental Information Processing:1; Organismal Systems:1
AM PRO	120	19	5	18	out (63)	All (8261)	Human Diseases:34; Organismal Systems:32; Metabolism:30; Cellular Processes:12; Environmental Information Processing:9; Genetic Information Processing:3
VM PEP	14	4	0	2	out (3)	All (108)	Metabolism:7; Human Diseases:4; Organismal Systems:2; Genetic Information Processing:1
VM PRO	182	33	16	43	out (94)	All (3519)	Human Diseases:48; Metabolism:47; Organismal Systems:45; Environmental Information Processing:17; Cellular Processes:16; Genetic Information Processing:9

S6_KEGG_top_pathways

g	keg	kegg	path	ou	back	pva	qva	pat	genes

roup	g_a_class	_b_class	way	t_count	ground_count	lue	lue	hway_id	
AMPEP	Human Diseases	Infectious diseases	Hepatitis B	1	3	0.0441128	0.0781279	ko05161	XP_003249259.2
AMPEP	Metabolism	Nucleotide metabolism	Purine metabolism	1	4	0.0585959	0.0781279	ko00230	XP_001121462.1
AMPEP	Metabolism	Nucleotide metabolism	Pyrimidine metabolism	1	4	0.0585959	0.0781279	ko00240	XP_001121462.1
AMPEP	Genetic Information Processing	Transcription	RNA polymerase	1	4	0.0585959	0.0781279	ko03020	XP_001121462.1
AMPEP	Environmental Information Processing	Signal transduction	mTOR signaling pathway	1	4	0.0585959	0.0781279	ko04150	XP_003249259.2
AMPEP	Organismal Systems	Immune system	Cytosolic DNA-sensing pathway	1	4	0.0585959	0.0781279	ko04623	XP_001121462.1
AM	Human	Infectious	Epstein-	1	5	0.0729	0.0833	ko05	XP_001121462.1

PEP	Diseases	diseases	Barr virus infection			685	926	169	
AMPEP	Metabolism	Global and overview maps	Metabolic pathways	1	39	0.495854	0.495854	ko01100	XP_001121462.1
AMPRO	Genetic Information Processing	Folding, sorting and degradation	Protein processing in endoplasmic reticulum	15	202	1.72798e-11	2.07358e-09	ko04141	XP_006567277.1;XP_026296867.1;XP_026299043.1;XP_006565093.1;XP_006568873.1;XP_026298883.1;XP_006569897.2;NP_001153524.1;XP_395614.3;XP_006564557.1;XP_625125.2;XP_006559569.1;XP_623282.2;XP_623603.1;XP_026296268.1
AMPRO	Cellular Processes	Transport and catabolism	Lysosome	12	173	5.12559e-09	3.07535e-07	ko04142	XP_001120140.3;XP_624310.2;XP_006562719.1;XP_001121565.2;XP_006570673.1;XP_001121483.3;XP_392857.2;XP_392381.3;XP_395852.4;XP_026297212.1;NP_001011619.1;XP_026300654.1
AMPRO	Organismal Systems	Immune system	Antigen processing and presentation	5	21	4.06623e-07	1.62649e-05	ko04612	NP_001153524.1;XP_016770902.1;XP_624977.1;XP_006559569.1;XP_623282.2
AMPRO	Human Diseases	Neurodegenerative diseases	Prion diseases	3	23	0.000671433	0.020143	ko05020	NP_001153524.1;XP_026299738.1;NP_001171498.1
AMPRO	Metabolism	Glycan biosynthesis and metabolism	Glycosaminoglycan degradation	3	31	0.00163174	0.0391619	ko00531	XP_001121565.2;NP_001011619.1;XP_026300654.1
A	Met	Glyc	Other	3	38	0.0	0.0	ko	XP_001121565.2;XP_395852.4;XP_026297212.1

M PRO	abolism	an biosynthesis and metabolism	glycan degradation			0294843	589687	00511	
AM PRO	Metabolism	Amino acid metabolism	Glycine, serine and threonine metabolism	3	54	0.00795175	0.135058	ko00260	XP_016767722.1;XP_394209.2;XP_394210.5
AM PRO	Metabolism	Lipid metabolism	Linoleic acid metabolism	2	19	0.00900385	0.135058	ko00591	NP_001011614.1;XP_001120293.2
AM PRO	Metabolism	Lipid metabolism	alpha-Linolenic acid metabolism	2	21	0.0109497	0.145997	ko00592	NP_001011614.1;XP_001120293.2
AM PRO	Organismal Systems	Endocrine system	Renin-angiotensin system	2	28	0.019048	0.228575	ko04614	XP_006558237.1;XP_623670.3
AM PRO	Organismal Systems	Digestive system	Protein digestion and absorption	4	152	0.0283876	0.243529	ko04974	XP_016772200.1;XP_026296056.1;XP_623670.3;XP_026299326.1
AM PR	Organismal Systems	Digestive system	Fat digestion and absor	3	90	0.0310581	0.243529	ko04975	NP_001119716.1;NP_001011614.1;XP_001120293.2

O			ption						
VM PEP	Metabolism	Carbohydrate metabolism	Citrate cycle (TCA cycle)	1	1	0.0277778	0.0972222	ko00020	Gene.59::TRINITY_DN10461_c0_g1::g.59::m.59
VM PEP	Metabolism	Carbohydrate metabolism	Pyruvate metabolism	1	1	0.0277778	0.0972222	ko00620	Gene.59::TRINITY_DN10461_c0_g1::g.59::m.59
VM PEP	Metabolism	Carbohydrate metabolism	Glyoxylate and dicarboxylate metabolism	1	1	0.0277778	0.0972222	ko00630	Gene.59::TRINITY_DN10461_c0_g1::g.59::m.59
VM PEP	Metabolism	Global and overview maps	Carbon metabolism	1	1	0.0277778	0.0972222	ko01200	Gene.59::TRINITY_DN10461_c0_g1::g.59::m.59
VM PEP	Metabolism	Amino acid metabolism	Cysteine and methionine metabolism	1	2	0.0550363	0.154102	ko00270	Gene.59::TRINITY_DN10461_c0_g1::g.59::m.59
VM PEP	Organismal Systems	Nervous system	Retrograde endocannabinoid signaling	1	3	0.0817806	0.190821	ko04723	Gene.2930::TRINITY_DN13078_c0_g4::g.2930::m.2930
VM PEP	Genetic Information Pro	Translation	Ribosome	1	4	0.108015	0.214331	ko03010	Gene.585::TRINITY_DN12396_c0_g2::g.585::m.585

	cessing								
VM PEP	Human Diseases	Endocrine and metabolic diseases	Non-alcoholic fatty liver disease (NAFLD)	1	5	0.133746	0.214331	ko04932	Gene.2930::TRINITY_DN13078_c0_g4::g.2930::m.2930
VM PEP	Metabolism	Global and overview maps	Metabolic pathways	2	27	0.153588	0.214331	ko01100	Gene.2930::TRINITY_DN13078_c0_g4::g.2930::m.2930;Gene.59::TRINITY_DN10461_c0_g1::g.59::m.59
VM PEP	Metabolism	Energy metabolism	Oxidative phosphorylation	1	6	0.158976	0.214331	ko00190	Gene.2930::TRINITY_DN13078_c0_g4::g.2930::m.2930
VM PEP	Organismal Systems	Environmental adaptation	Thermogenesis	1	7	0.183713	0.214331	ko04714	Gene.2930::TRINITY_DN13078_c0_g4::g.2930::m.2930
VM PEP	Human Diseases	Neurodegenerative diseases	Parkinson disease	1	7	0.183713	0.214331	ko05012	Gene.2930::TRINITY_DN13078_c0_g4::g.2930::m.2930
VM PRO	Organismal Systems	Immune system	Antigen processing and presentation	7	18	1.94238e-07	3.53514e-05	ko04612	Gene.3563::TRINITY_DN13203_c2_g2::g.3563::m.3563;Gene.3090::TRINITY_DN13107_c1_g11::g.3090::m.3090;Gene.3189::TRINITY_DN13122_c1_g2::g.3189::m.3189;Gene.7747::TRINITY_DN14081_c1_g8::g.7747::m.7747;Gene.6523::TRINITY_DN13817_c2_g8::g.6523::m.6523;Gene.14944::TRINITY_DN15571_c2_g1::g.14944::m.14944;Gene.15771::TRINITY_DN15718_c1_g9::g.15771::m.15771
VM PRO	Cellular Processes	Transport and catabolism	Lysosome	13	94	7.77862e-07	5.04904e-05	ko04142	Gene.14138::TRINITY_DN15410_c0_g5::g.14138::m.14138;Gene.5145::TRINITY_DN13534_c3_g14::g.5145::m.5145;Gene.13010::TRINITY_DN15192_c1_g13::g.13010::m.13010;Gene.5435::TRINITY_DN13601_c1_g7::g.5435::m.5435;Gene.14331::TRINITY_DN15446_c1_g9::g.14331::m.14331;Gene.16501::TRINITY_DN15820_c3_g22::g.16501::m.16501;Gene.9384::TRINITY_DN14438_c3_g4::g.93...
V	Met	Glob	Meta	43	814	8.3	5.0	ko	Gene.15196::TRINITY_DN15617_c0_g3::g.15196::m.15196;Gene.14440::TRINITY_DN15472_c0_g1::g.14440::m.1444

M PRO	abolism	al and overview maps	bolic pathways			226e-07	4904e-05	01100	0;Gene.12239::TRINITY_DN15036_c0_g10::g.12239::m.12239;Gene.5145::TRINITY_DN13534_c3_g14::g.5145::m.5145;Gene.13010::TRINITY_DN15192_c1_g13::g.13010::m.13010;Gene.14331::TRINITY_DN15446_c1_g9::g.14331::m.14331;Gene.13895::TRINITY_DN15360_c1_g3::...
VM PRO	Metabolism	Glycan biosynthesis and metabolism	Other glycan degradation	6	18	4.43711e-06	0.000201888	ko00511	Gene.5145::TRINITY_DN13534_c3_g14::g.5145::m.5145;Gene.13010::TRINITY_DN15192_c1_g13::g.13010::m.13010;Gene.5435::TRINITY_DN13601_c1_g7::g.5435::m.5435;Gene.14331::TRINITY_DN15446_c1_g9::g.14331::m.14331;Gene.4222::TRINITY_DN13334_c1_g15::g.4222::m.4222;Gene.12909::TRINITY_DN15172_c2_g1::g.12909::m.12909
VM PRO	Metabolism	Carbohydrate metabolism	Glycolysis / Gluconeogenesis	7	39	5.93206e-05	0.0215927	ko00010	Gene.1762::TRINITY_DN12820_c1_g1::g.1762::m.1762;Gene.775::TRINITY_DN12569_c0_g1::g.775::m.775;Gene.9728::TRINITY_DN14519_c1_g1::g.9728::m.9728;Gene.799::TRINITY_DN12586_c0_g1::g.799::m.799;Gene.12728::TRINITY_DN15134_c4_g5::g.12728::m.12728;Gene.637::TRINITY_DN12450_c0_g1::g.637::m.637;Gene.11221::TRINITY_DN14818_c2_g3::g.11221::m.11221
VM PRO	Metabolism	Carbohydrate metabolism	Starch and sucrose metabolism	5	18	7.95326e-05	0.0241249	ko00500	Gene.14440::TRINITY_DN15472_c0_g1::g.14440::m.14440;Gene.1655::TRINITY_DN12800_c1_g14::g.1655::m.1655;Gene.1802::TRINITY_DN12829_c0_g3::g.1802::m.1802;Gene.15358::TRINITY_DN15648_c1_g1::g.15358::m.15358;Gene.8750::TRINITY_DN14297_c1_g15::g.8750::m.8750
VM PRO	Metabolism	Carbohydrate metabolism	Amino sugar and nucleotide sugar metabolism	7	49	0.000266384	0.0692599	ko00520	Gene.5145::TRINITY_DN13534_c3_g14::g.5145::m.5145;Gene.13010::TRINITY_DN15192_c1_g13::g.13010::m.13010;Gene.1202::TRINITY_DN12702_c0_g13::g.1202::m.1202;Gene.1206::TRINITY_DN12702_c0_g2::g.1206::m.1206;Gene.12799::TRINITY_DN15149_c1_g7::g.12799::m.12799;Gene.13945::TRINITY_DN15367_c2_g6::g.13945::m.13945;Gene.15358::TRINITY_DN15648_c1_g1::g.1535...
VM PRO	Metabolism	Glycan biosynthesis and metabolism	Glycosaminoglycan degradation	5	24	0.000347421	0.0790382	ko00531	Gene.5145::TRINITY_DN13534_c3_g14::g.5145::m.5145;Gene.13010::TRINITY_DN15192_c1_g13::g.13010::m.13010;Gene.14331::TRINITY_DN15446_c1_g9::g.14331::m.14331;Gene.7375::TRINITY_DN14006_c2_g5::g.7375::m.7375;Gene.11325::TRINITY_DN14842_c2_g3::g.11325::m.11325
VM	Metabolism	Glycan biosy	Glycosphingoli	3	7	0.0005978	0.0108807	ko0060	Gene.5145::TRINITY_DN13534_c3_g14::g.5145::m.5145;Gene.13010::TRINITY_DN15192_c1_g13::g.13010::m.13010;Gene.10266::TRINITY_DN14626_c0_g5::g.10266::m.10266

PRO		nthesis and metabolism	pid biosynthesis - globo and isoglobo series			38		3	
VM PRO	Metabolism	Glycan biosynthesis and metabolism	Glycosphingolipid biosynthesis - ganglio series	3	7	0.000597838	0.0108807	ko00604	Gene.5145::TRINITY_DN13534_c3_g14::g.5145::m.5145;Gene.13010::TRINITY_DN15192_c1_g13::g.13010::m.13010;Gene.14331::TRINITY_DN15446_c1_g9::g.14331::m.14331
VM PRO	Organismal Systems	Endocrine system	Estrogen signaling pathway	6	49	0.0017131 1	0.0283443	ko04915	Gene.16375::TRINITY_DN15807_c1_g4::g.16375::m.16375;Gene.6523::TRINITY_DN13817_c2_g8::g.6523::m.6523;Gene.14944::TRINITY_DN15571_c2_g1::g.14944::m.14944;Gene.15771::TRINITY_DN15718_c1_g9::g.15771::m.15771;Gene.2210::TRINITY_DN12923_c0_g2::g.2210::m.2210;Gene.9384::TRINITY_DN14438_c3_g4::g.9384::m.9384
VM PRO	Human Diseases	Neurodegenerative diseases	Prion diseases	4	21	0.0020164 2	0.0291288	ko05020	Gene.3563::TRINITY_DN13203_c2_g2::g.3563::m.3563;Gene.2325::TRINITY_DN12952_c1_g2::g.2325::m.2325;Gene.16528::TRINITY_DN15822_c3_g16::g.16528::m.16528;Gene.15771::TRINITY_DN15718_c1_g9::g.15771::m.15771

Table S7. STRING/Cytoscape network summaries and representative edges

S7_STRING_summary

group	nodes	edges	mean_combined_score	max_combined_score	top_degree_nodes
AM PEP	3	3	0.558	0.63	Melt:2; Mcdp:2; LOC406145:2
AM PRO	70	150	0.6503	0.999	Crc:18; Hsc70-3:17; ERp60:16; Pdi:15; LOC412150:11
VM PEP	0	0			
VM PRO	93	213	0.6302	0.997	XP_003490898.1:19; XP_003488962.1:16; XP_003491978.1:16; XP_003486363.1:15; XP_003484418.1:14

S7_STRING_top_edges

group	node1	node2	combined_score	coexpression	experimental	textmining
AM PEP	Melt	Mcdp	0.63	0	0	0.63
AM PEP	LOC406145	Mcdp	0.538	0	0	0.538
AM PEP	LOC406145	Melt	0.506	0	0	0.506
AM PRO	ERp60	Crc	0.999	0.965	0.699	0.927
AM PRO	Hsc70-3	LOC412150	0.999	0.993	0.33	0.915
AM PRO	LOC552747	LOC551205	0.998	0.122	0.861	0.904
AM PRO	LOC412150	Crc	0.998	0.972	0.794	0.812
AM PRO	LOC551763	Hsc70-3	0.997	0.988	0.655	0.905
AM PRO	LOC551763	LOC412150	0.997	0.987	0.237	0.807
AM PRO	Pdi	Crc	0.993	0.743	0.692	0.92
AM PRO	LOC552223	LOC412150	0.987	0.948	0.14	0.748
AM PRO	Hsc70-3	Pdi	0.985	0.715	0.353	0.926
AM PRO	ERp60	LOC412150	0.98	0.895	0.355	0.734
VM PRO	XP_003493872.1	XP_003490898.1	0.997	0.963	0.391	0.913
VM PRO	XP_003491320.1	XP_003486688.1	0.996	0.963	0.905	0
VM PRO	XP_003492045.1	XP_003486363.1	0.995	0.872	0.66	0.914
VM PRO	XP_003493872.1	XP_003486363.1	0.995	0.915	0.759	0.819
VM PRO	XP_003486363.1	XP_003484418.1	0.992	0.78	0.652	0.914
VM PRO	XP_003491166.1	XP_003490305.1	0.992	0.702	0.198	0.697
VM PRO	XP_003486045.1	XP_003486043.1	0.992	0.987	0.19	0.915
VM PRO	XP_003492846.1	XP_003488962.1	0.991	0.391	0	0.878
VM PRO	XP_003492846.1	XP_003490305.1	0.986	0.551	0	0.722
VM PRO	XP_003491978.1	XP_003489162.1	0.984	0.552	0.371	0.409

Table S8. Transcriptome sequencing, assembly, and annotation summary

S8_transcriptome_summary

metric	value
sample_groups	Y1:3; Y2:3; Y3:3; Y4:3; Y5:3
Valid_Reads_min_mean_max	38625426.0; 47285416.933; 54617800.0
Valid_Bases_min_mean_max	5.24; 6.419; 7.42
Valid%_min_mean_max	92.12; 93.793; 95.3
Q20%_min_mean_max	97.44; 97.521; 97.64
Q30%_min_mean_max	92.4; 92.558; 92.79
GC%_min_mean_max	41.55; 42.347; 43.21
Transcript_All	81673
Transcript_GC%	38.59
Transcript_Min Length	201
Transcript_Median Length	458
Transcript_Max Length	23092
Transcript_Total Assembled Bases	69874718
Transcript_N50	1526
Gene_All	35752
Gene_GC%	36.68
Gene_Min Length	201
Gene_Median Length	370
Gene_Max Length	23092
Gene_Total Assembled Bases	25470269
Gene_N50	1236
assembled_genes	35752
Annotation_nonblank	11025
Name_nonblank	6844
GO_Term_nonblank	7880
KOEntry_nonblank	7899
KEGG_Pathway_nonblank	7899
Pfam_description_nonblank	7953

Additional supporting table. Sample quantification records

QC_sample_quantification

table	metric	sample	concentration_ug_per_uL	volume_uL	total_ug
protein_amount	protein amount	VM-V pro	22.99	200	4598
protein_amount	protein amount	AM-V pro	12.86	200	2572
peptide_amount	peptide amount	VM-V pro	0.54	80	43.2
peptide_amount	peptide amount	AM-V pro	0.721	80	57.68
peptide_amount	peptide amount	VM-V pep	2.413	100	241.3
peptide_amount	peptide amount	AM-V pep	1.935	100	193.5

Additional supporting table. FASTA record summary

FASTA_record_summary

fasta	records	min_length	median_length	max_length
AM_pro_deps.fasta	164	46	395.5	4483
VM_pro_deps.fasta	196	103	340	3353
AM_PEP_DEPS.fasta	11	50	91	109
VM_PEP_deps.fasta	11	99	103	108
